# Effects of Hospital-Based Comprehensive Medication Reviews Including Postdischarge Follow-up on Older Patients’ Use of Health Care: A Cluster Randomized Clinical Trial

**DOI:** 10.1001/jamanetworkopen.2021.6303

**Published:** 2021-04-30

**Authors:** Thomas G. H. Kempen, Maria Bertilsson, Nermin Hadziosmanovic, Karl-Johan Lindner, Håkan Melhus, Elisabet I. Nielsen, Johanna Sulku, Ulrika Gillespie

**Affiliations:** 1 Hospital Pharmacy Department, Uppsala University Hospital, Uppsala, Sweden; 2 Department of Medical Sciences, Uppsala University, Uppsala, Sweden; 3 Uppsala Clinical Research Center, Uppsala, Sweden; 4 Pharmacy Department, Region Västmanland, Västerås, Sweden; 5 Department of Pharmacy, Uppsala University, Uppsala, Sweden; 6 Pharmacy Department, Region Gävleborg, Gävle, Sweden; 7 Centre for Research and Development, Uppsala University/Region Gävleborg, Gävle, Sweden

## Abstract

**Importance:**

Suboptimal use of medications is a leading cause of health care–related harm. Medication reviews improve medication use, but evidence of the possible benefit of inpatient medication review for hard clinical outcomes after discharge is scarce.

**Objective:**

To study the effects of hospital-based comprehensive medication reviews (CMRs), including postdischarge follow-up of older patients’ use of health care resources, compared with only hospital-based reviews and usual care.

**Design, Setting, and Participants:**

The Medication Reviews Bridging Healthcare trial is a cluster randomized crossover trial that was conducted in 8 wards with multiprofessional teams at 4 hospitals in Sweden from February 6, 2017, to October 19, 2018, with 12 months of follow-up completed December 6, 2019. The study was prespecified in the trial protocol. Outcome assessors were blinded to treatment allocation. In total, 2644 patients aged 65 years or older who had been admitted to 1 of the study wards for at least 1 day were included. Data from the modified intention-to-treat population were analyzed from December 10, 2019, to September 9, 2020.

**Interventions:**

Each ward participated in the trial for 6 consecutive 8-week periods. The wards were randomized to provide 1 of 3 treatments during each period: CMR, CMR plus postdischarge follow-up, and usual care without a clinical pharmacist.

**Main Outcomes and Measures:**

The primary outcome measure was the incidence of unplanned hospital visits (admissions plus emergency department visits) within 12 months. Secondary outcomes included medication-related admissions, visits with primary care clinicians, time to first unplanned hospital visit, mortality, and costs of hospital-based care.

**Results:**

Of the 2644 participants, 7 withdrew after inclusion, leaving 2637 for analysis (1357 female [51.5%]; median age, 81 [interquartile range, 74-87] years; median number of medications, 9 [interquartile range, 5-13]). In the modified intention-to-treat analysis, 922 patients received CMR, 823 received CMR plus postdischarge follow-up, and 892 received usual care. The crude incidence rate of unplanned hospital visits was 1.77 per patient-year in the total study population. The primary outcome did not differ between the intervention groups and usual care (adjusted rate ratio, 1.04 [95% CI, 0.89-1.22] for CMR and 1.15 [95% CI, 0.98-1.34] for CMR plus postdischarge follow-up). However, CMR plus postdischarge follow-up was associated with an increased incidence of emergency department visits within 12 months (adjusted rate ratio, 1.29; 95% CI, 1.05-1.59) compared with usual care. There were no differences between treatment groups regarding other secondary outcomes.

**Conclusions and Relevance:**

In this study of older hospitalized patients, CMR plus postdischarge follow-up did not decrease the incidence of unplanned hospital visits. The findings do not support the performance of hospital-based CMRs as conducted in this trial. Alternative forms of medication reviews that aim to improve older patients’ health outcomes should be considered and subjected to randomized clinical trials.

**Trial Registration:**

ClinicalTrials.gov Identifier: NCT02986425

## Key Points

**Question:**

What are the effects of hospital-based comprehensive medication reviews (CMRs) that include postdischarge follow-up of older patients’ use of health care resources?

**Findings:**

In this cluster randomized crossover trial that included 2637 older hospitalized patients in Sweden, the incidence of unplanned hospital visits within 12 months did not differ between patients who received a CMR that included postdischarge follow-up compared with those who received either a hospital-based CMR alone or usual care.

**Meaning:**

Hospital-based CMRs, including postdischarge follow-up as conducted in this trial, did not decrease the incidence of unplanned hospital visits in older patients.

## Teaser

This cluster randomized crossover trial assesses the effects of hospital-based comprehensive medication reviews, including postdischarge follow-up of older patients’ use of health care resources, compared with only hospital-based reviews and usual care.

## Introduction

Suboptimal use of medications is a leading cause of avoidable harm in health care systems around the world.^1,2^ Medication-related harm may often arise as a consequence of medication errors occurring at transitions of care.^3,4^ In response, the World Health Organization established a global patient safety initiative to reduce severe, avoidable medication-related harm by 50% from 2017 to 2022.^5^ One of the proposed actions is to perform medication reviews in patients who use multiple medications.^6^

A medication review can be interpreted in different ways, but it is often defined as a structured, critical examination of a person’s medications with the objective of reaching an agreement with the person about treatment, optimizing the impact of medications, and minimizing medication-related harm.^7^ Medication reviews can range from a paper-based review of a patient’s medication list by a single clinician to a more comprehensive, multiprofessional approach that directly involves patients and takes their underlying conditions and symptoms into account.^8^ Medication reviews in hospitalized patients have been shown to decrease medication-related problems and inappropriate prescribing, but it is unclear whether these improvements in medication treatment also affect hard clinical end points after discharge.^9,10^ A 2016 Cochrane review^10^ expressed the need for high-quality trials that incorporate at least 1 year of follow-up and are randomized at a cluster level to minimize contamination bias. A cluster randomized crossover design, in which each cluster is randomly assigned to a sequence of interventions, could also improve statistical efficiency and reduce the required number of clusters.^11^

In a previous randomized clinical trial (RCT)^12^ conducted at Uppsala University Hospital, Sweden, in 2005-2006, patients 80 years or older who received a comprehensive medication review (CMR) that included follow-up after hospital discharge performed by a pharmacist as part of the ward team had a 16% reduction in hospital visits the following year. Medication-related readmissions were reduced by 80%.^12^ In a recent multicenter RCT among older patients in Denmark,^13^ hospital-based medication reviews with motivational interviewing on discharge and postdischarge follow-up performed by a ward-based pharmacist resulted in a 25% reduction in the risk of being readmitted within 6 months compared with usual care. In recent decades, Swedish health care authorities have taken various measures to improve the quality of care for older patients, such as developing quality indicators, educating health care professionals in geriatric pharmacology, and funding local initiatives.^14,15^ In this context, numerous hospitals in Sweden have used ward-based pharmacists who perform daily medication reviews in older patients. However, the resources allocated for structured provision of CMRs—including postdischarge follow-up—are insufficient, as studied in previous trials.^12,13^ It is currently unclear which forms of medication reviews are most beneficial to patients. Pragmatic trials, which are designed to show the real-world effectiveness of clinical interventions, may enable prioritization of interventions and patients in clinical practice.^16^ The goal of this pragmatic cluster randomized crossover trial (the Medication Reviews Bridging Healthcare [MedBridge] trial) was to compare the effects of hospital-based CMRs, including postdischarge follow-up on older patients’ use of health care resources, with those of hospital-based reviews only and usual care.

## Methods

### Study Design and Participants

The MedBridge trial is a pragmatic, multicenter, cluster randomized crossover trial that incorporated 3 treatments. The trial was performed and is reported in accordance with the Helsinki Declaration^17^ and the applicable extensions of the Consolidated Standards of Reporting Trials (CONSORT) reporting guideline.^18,19,20^ The trial received ethical approval from the Swedish Central Ethical Review Board. All participants provided written informed consent or verbal informed consent if written informed consent was not possible. The rationale and design of the trial have been described previously.^21^ The study was prespecified in the trial protocol, which is provided in Supplement 1; the statistical analysis plan is provided in Supplement 2. A process evaluation was conducted alongside the trial to understand how the interventions were implemented and performed and which factors may have affected the trial’s results. Findings from this process evaluation have been published elsewhere.^22,23,24^

**Figure 1.**
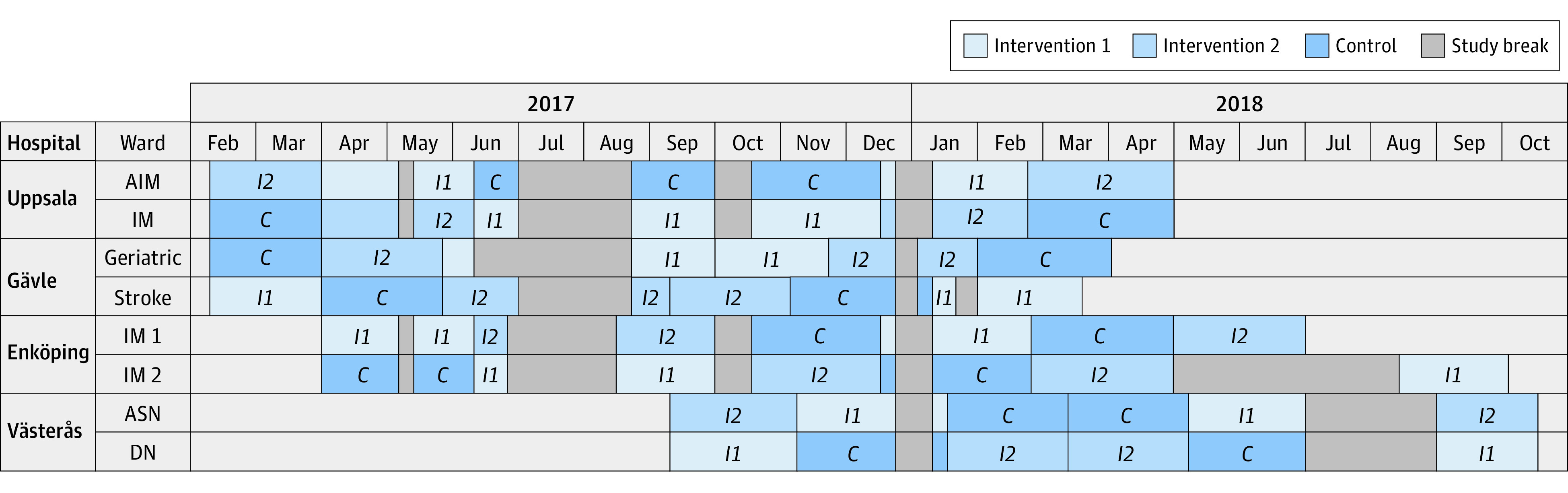
Ward (Cluster) and Period Chart as Randomized and Performed in the Medication Reviews Bridging Healthcare Trial AIM indicates acute internal medicine; ASN, acute stroke and neurology; DN, diabetes and nephrology; and IM, internal medicine.

The trial was conducted from February 6, 2017, until October 19, 2018, at 4 hospitals in Sweden (Figure 1). Two wards per hospital were included, with each ward acting as a cluster. The 8 wards differed in terms of medical specialty: internal medicine (n = 3), acute internal medicine (n = 1), acute stroke and neurology (n = 1), diabetes and nephrology (n = 1), geriatrics (n = 1), and stroke (n = 1). The selection of these wards was based on the high prevalence of older patients using multiple medications as well as the fact that the performance of medication reviews by ward-based pharmacists was an established practice or that pharmacists had been introduced at each ward at least 6 months before the start of the trial. The pharmacists (including T.G.H.K. and J.S.) had either completed a full-time, 1-year postgraduate program in clinical pharmacy or taken undergraduate courses in clinical pharmacy and advanced pharmacotherapy in Sweden. All pharmacists had at least 6 months of experience performing medication reviews in a multiprofessional ward team. To assess clinical skill performance in relation to medication reviews, all pharmacists participated in an objective structured clinical examination before the start of the trial. Two training days were held, one before and the other during the trial, with patient case discussions among the pharmacists to ensure common practice across all sites. Other ward staff did not receive additional training.

All patients who were eligible for inclusion during the trial were asked to provide informed consent to participate. Inclusion criteria were age 65 years or older and admission to one of the participating wards for at least one 24-hour period on regular weekdays. Patients were excluded if they had undergone a medication review by a clinical pharmacist within the last 30 days, as stated in their electronic health record (EHR), did not reside in the hospital’s county, or were receiving palliative treatment, as stated in their EHR. Medication reconciliation (ie, the process of ensuring an accurate and complete medication list^25^) by a pharmacist at the emergency department (ED) was not considered an exclusion criterion.

### Randomization and Masking

Crossover and randomization took place at a cluster (ie, ward) level within each hospital. Each ward participated in the trial for 6 consecutive 8-week study periods, which were divided into 2 separate blocks of 3 study periods each. During each period, 1 of 3 treatments (intervention 1 or 2 or control) was provided at the ward, with permuted block randomization ensuring that each treatment was performed within each block (Figure 1). Details of the block randomization are available in eMethods 1 in Supplement 3.

Participants and ward staff were not blinded to treatment allocation. All patients who were eligible for inclusion received the treatment that was being performed at the ward at that time, regardless of informed consent to participate in the trial. Individual patients could only be included in the trial once. Any participant who was readmitted to one of the study wards also received the intervention being performed at that time.

### Interventions

The different interventions pertained to individual patients. Intervention 1, CMR, consisted of a medication reconciliation by a clinical pharmacist with the patient, next-of-kin, or caregiver within 24 hours after hospital admission to ensure a correct list of medications. This reconciliation was directly followed by a CMR in collaboration with the ward physician, the nurse if appropriate, and the patient, next-of-kin, or caregiver if possible, similar to a level 3 medication review as described in the literature.^8^ A level 3 medication review includes a structured, critical examination of the patient’s medications in relation to the patient’s symptoms and conditions, based on information from the patient and the EHR. The objective was to reach an agreement about the continued appropriateness and effectiveness of the treatment, including treatment not related to the cause of admission and untreated indications. Other issues, such as adherence, practical use of medications (eg, how to use an inhaler), dosage forms, adverse effects, interactions, and the patient’s understanding of their condition and its treatment, were considered when appropriate. The effects of medication changes resulting from the CMR were monitored during the hospital stay by the physician or pharmacist. On discharge, a medication reconciliation was performed by the pharmacist to ensure that the patient’s medication list and prescriptions for medications to be used after the hospital stay were consistent with the patient’s EHR. This medication reconciliation did not necessarily involve contact with the patient or other ward staff.

Intervention 2, a hospital-based CMR that included postdischarge follow-up (CMR plus follow-up), consisted of the same elements as intervention 1 with the following additions: a referral sent by the clinical pharmacist in consultation with the ward physician to the patient’s primary care clinician with recommendations on monitoring needs or actions to be taken after hospital discharge, if deemed necessary. A first telephone call to the patient, next-of-kin, or caregiver by the pharmacist 2 to 7 days after discharge ensured that the information about the patient’s current medication treatment had been understood correctly and addressed any related problems, concerns, or questions. A second telephone call by the pharmacist 1 to 2 months after discharge inquired how the patient was managing the medications and whether any problems, concerns, or questions had arisen. Counseling was provided, and any other necessary actions were taken by the pharmacist.

Control patients received usual hospital care. According to Swedish regulations, usual care should include medication reconciliation on admission for patients who are 75 years or older and are taking 5 or more medications, followed by a medication review if medication-related problems are present.^26^ Adherence to these regulations in hospital practice seems generally low.^27^ Some of the intervention components or related activities could thus be performed to a certain degree, but no pharmacist was involved at the ward. In contrast, approximately 1 full-time-equivalent clinical pharmacist was available per ward during each intervention period (CMR or CMR plus follow-up).

### Outcomes, Data Collection, and Sample Size

The primary outcome measure was the incidence of all-cause unplanned hospital visits (admissions plus visits to the ED) within 12 months after the index admission. An unplanned visit was defined as a visit that had not been part of the patient’s treatment plan but resulted from an acute health problem. Secondary outcomes were all-cause unplanned hospital admissions and separate ED visits, unplanned medication-related admissions, visits with a primary care clinician, time to first unplanned hospital visit, all-cause mortality, and costs of hospital-based care. The primary outcome was also applied to predefined subgroups based on characteristics at index admission (baseline).

Baseline and outcome data were extracted from county EHR systems and health care registries. All primary and secondary outcome data collection and assessments were blinded to treatment allocation. A detailed description of the outcomes and data collection is provided in eMethods 1 in Supplement 3. According to previous power simulations^21^ described in eMethods 1 in Supplement 3, 2310 study participants would be needed in total to show a 10% reduction of unplanned hospital visits between the CMR plus follow-up group and the usual care group with a power of approximately 83%.

### Statistical Analysis

Data from the modified intention-to-treat population were analyzed from December 10, 2019, to September 9, 2020. Primary and secondary outcome analyses were based on a modified intention-to-treat (ITT) population. The ITT population was defined as all patients who were eligible for inclusion in the trial. Patients who provided informed consent were considered part of the modified ITT population, thus representing all participants who could be included in the analyses. Patients who did not provide informed consent and those who withdrew before data collection were considered dropouts. The ITT population was used for sensitivity analysis of the primary outcome measure, because multiple imputation was needed for patients without consent to participate (see eMethods 2, eTable 1, and eTable 2 in Supplement 3 for detailed procedures and results).

The differences in incidence rates of visits and admissions between treatment groups were compared using log-linear models with Poisson variance function, with adjustments made for cluster and period effects. The number of unplanned hospital visits within 12 months before the index admission was used as a patient-level covariate, and the number of out-of-hospital days was used as an offset. Tukey adjusted *P* values and 95% CIs were calculated to prevent multiple testing problems. Time to first unplanned hospital visit and mortality were analyzed with nested frailty models to account for the hierarchical clustering of the data (hospital ward). The nonparametric bootstrap method was used to compare costs of hospital-based care and to estimate 95% CIs. A statistical significance level of *P* < .05 (2-tailed) was used for all comparisons. All analyses were performed using SAS software, version 9.4 (SAS Institute Inc). Statisticians were blinded to treatment allocation until database closure. Full details are provided in eMethods 1 in Supplement 3.

## Results

**Figure 2.**
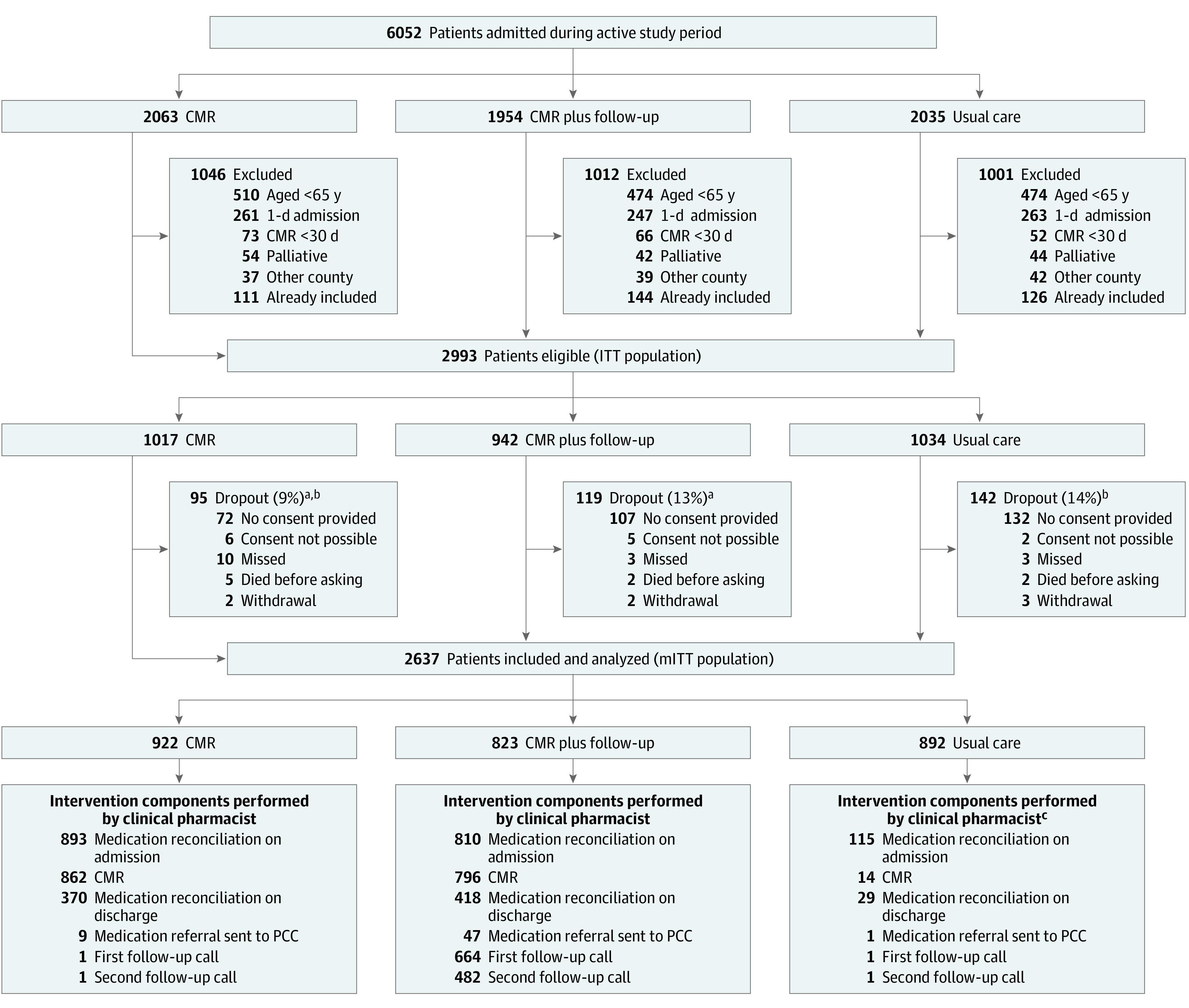
CONSORT Diagram of the Medication Reviews Bridging Healthcare Trial CMR indicates comprehensive medication review; CONSORT, Consolidated Standards of Reporting Trials; ITT, intention-to-treat; mITT, modified ITT; and PCC, primary care clinician. ^a^*P* = .02, χ^2^ test for differences in dropouts between CMR and usual care groups. ^b^*P* < .01, χ^2^ test for differences in dropouts between CMR plus follow-up and usual care groups. ^c^Indicates a protocol violation.

In total, 6052 patients were admitted during the study periods (Figure 2). All patients were screened for eligibility. Of the 2993 eligible patients, 2644 (88%) provided written informed consent to participate. Seven participants withdrew their consent before data collection. The percentage of dropouts differed between CMR (95 of 1017 [9.3%]) and CMR plus follow-up (119 of 942 [12.6%]; *P* = .02) and between CMR and usual care (142 of 1034 [13.7%]; *P* = .002). A total of 2637 participants (922 in CMR, 823 in CMR plus follow-up, and 892 in usual care groups) were included in the modified ITT analysis, with 356 eligible patients (11.9%) not included.

Participants in each treatment group were similar in terms of baseline characteristics (Table 1).^47,48^ The median age was 81 (interquartile range, 74-87) years, 1357 (51.5%) were female and 1280 (48.5%) were male, and a median of 9 (interquartile range, 5-13) medications were prescribed. The treatment group sizes within each cluster ranged from 63 to 146 participants.

**Table 1.** Modified ITT Population Baseline Characteristics

Characteristic	Treatment group^a^		
	CMR (n = 922)	CMR plus follow-up (n = 823)	Usual care (n = 892)
Age, median (IQR), y	81 (74-87)	81 (74-87)	80 (74-87)
Sex			
Female	458 (49.7)	452 (54.9)	447 (50.1)
Male	464 (50.3)	371 (45.1)	445 (49.9)
eGFR <30 mL/min/1.73 m^2^^b^	183 (19.8)	139 (17.1)	155 (17.4)
No. of medications, median (IQR)	9 (5-13)	8 (5-13)	9 (6-13)
Automated drug-dispensing in home setting	244 (26.5)	218 (26.5)	216 (24.2)
Social support (home care or nursing home)	344 (37.3)	328 (39.9)	319 (35.8)
No. of unplanned hospital visits in 12 mo before inclusion, median (IQR)	1 (0-2)	1 (0-2)	1 (0-2)
5 most prevalent diagnoses in medical history			
Hypertension	649 (70.4)	572 (69.5)	605 (67.8)
Type 2 diabetes	281 (30.5)	214 (26.0)	252 (28.3)
Atrial fibrillation and flutter	265 (28.7)	225 (27.3)	235 (26.3)
Congestive heart failure	264 (28.6)	216 (26.2)	241 (27.0)
COPD	124 (13.4)	118 (14.3)	120 (13.5)
Age-adjusted CCI score, median (IQR)^c^	5 (4-7)	5 (4-6)	5 (4-6)
Hospital ward			
Internal medicine 1, Enköping	140 (15.2)	97 (11.8)	133 (14.9)
Internal medicine 2, Enköping	121 (13.1)	111 (13.5)	120 (13.5)
Stroke, Gävle	146 (15.8)	107 (13.0)	136 (15.2)
Geriatrics, Gävle	73 (7.9)	63 (7.7)	71 (8.0)
Acute internal medicine, Uppsala	115 (12.5)	102 (12.4)	108 (12.1)
Internal medicine, Uppsala	129 (14.0)	103 (12.5)	141 (15.8)
Acute stroke and neurology, Västerås	115 (12.5)	117 (14.2)	93 (10.4)
Diabetes and nephrology, Västerås	83 (9.0)	123 (14.9)	90 (10.1)

Abbreviations: CCI, Charlson Comorbidity Index; CMR, comprehensive medication review; COPD, chronic obstructive pulmonary disease; eGFR, estimated glomerular filtration rate; IQR, interquartile range; ITT, intention to treat.

^a^ Unless otherwise indicated, data are expressed as No. (%) of patients.

^b^ Patients with missing eGFR values were excluded from calculation, including 8 from the CMR plus follow-up and 1 from the usual care groups.

^c^ Based on registered diagnosis codes up to 2 years before index admission, classified in accordance with Quan et al,^47^ and calculated in accordance with Charlson et al.^48^

### Primary Outcome

The incidence of unplanned hospital visits within 12 months did not differ in the intervention groups compared with usual care: 1.74 visits for CMR (adjusted rate ratio [RR], 1.04; 95% CI, 0.89-1.22), 1.95 for CMR plus follow-up (adjusted RR, 1.15; 95% CI, 0.98-1.34), and 1.63 for usual care patients (Table 2); the crude incidence rate among all study participants was 1.77. There was no difference between groups in the primary outcome within 30 days, 3 months, or 6 months (eTable 3 in Supplement 3).

**Table 2.** Primary and Secondary Outcomes Within 12 Months

Outcome	Treatment group			Rate/hazard ratio (95% CI)			
				Crude		Adjusted^a^	
	CMR (n = 922)	CMR plus follow-up (n = 823)	Usual care (n = 892)	CMR vs usual care	CMR plus follow-up vs usual care	CMR vs usual care	CMR plus follow-up vs usual care
Crude rate^b^							
Unplanned hospital visits (primary outcome)	1.74	1.95	1.63	1.07 (0.99-1.15)	1.20 (1.12-1.29)	1.04 (0.89-1.22)	1.15 (0.98-1.34)
ED visits	0.84	0.97	0.71	1.18 (1.06-1.31)	1.36 (1.22-1.51)	1.16 (0.94-1.44)	1.29 (1.05-1.59)
Unplanned hospital admissions	0.89	0.98	0.91	0.98 (0.89-1.08)	1.08 (0.98-1.19)	0.95 (0.80-1.12)	1.04 (0.88-1.24)
Unplanned medication-related admissions	0.29	0.36	0.32	0.92 (0.78-1.09)	1.13 (0.96-1.33)	0.89 (0.69-1.16)	1.12 (0.87-1.45)
PCC visits	4.43	4.02	4.25	1.04 (1.00-1.09)	0.95 (0.90-0.99)	1.04 (0.91-1.19)	0.99 (0.86-1.15)
Time to first unplanned hospital visit, mean (SD), d^c^	203.2 (151.5)	201.8 (150.8)	208.1 (148.9)	1.02 (0.91-1.15)	1.03 (0.91-1.16)	1.03 (0.91-1.16)	1.05 (0.93-1.19)
All-cause mortality, No. (%)^c^	234 (25.4)	209 (25.4)	227 (25.4)	1.00 (0.83-1.20)	0.99 (0.82-1.20)	0.98 (0.81-1.18)	0.95 (0.79-1.15)
Costs of hospital-based care, mean (SD), $^d^	8987 (17 121)	9981 (18 963)	9901 (18 464)	NA^e^	NA^e^	NA	NA

Abbreviations: CMR, comprehensive medication review; ED, emergency department; NA, not applicable; PCC, primary care clinician.

^a^ Estimates are adjusted for cluster (ward) as random effect, study period as fixed effect, and unplanned hospital visits in 12 months before inclusion as patient-level covariate. Tukey’s adjusted 95% CIs are used for multiple comparisons.

^b^ Compared as rate ratios.

^c^ Compared as hazard ratios.

^d^ Including intervention costs: $58 for CMR and $94 for CMR plus follow-up. Costs are based on a Swedish krona to USD conversion rate of 0.11246 (as of January 1, 2019).

^e^ Difference of mean (95% CI based on 100 000 bootstrap estimates): CMR vs usual care, −$914 (−$2564 to $719); CMR plus follow-up vs usual care, $55 (−$1721 to $1823).

### Secondary Outcomes

There was no difference in the incidence of ED visits within 12 months between the CMR group (0.84 visits) and usual care (0.71 visits; adjusted RR, 1.16; 95% CI, 0.94-1.44) (Table 2). However, the incidence of ED visits within 12 months was increased in the CMR plus follow-up group (0.97 visits; adjusted RR, 1.29; 95% CI, 1.05-1.59) compared with usual care. The incidence of ED visits was also higher in the CMR plus follow-up group within 3 months (0.38 visits; adjusted RR, 1.39; 95% CI, 1.04-1.85) and 6 months (0.58 visits; adjusted RR, 1.27; 95% CI, 1.00-1.62) compared with usual care (eTable 3 in Supplement 3). No differences were seen in unplanned hospital admissions, unplanned medication-related admissions, visits with primary care clinicians, time to first unplanned hospital visit, all-cause mortality, or costs of hospital-based care for any of the follow-up periods (see Table 2 for 12-month follow-up and eTables 3 and 4 in Supplement 3 for 30-day, 3-month, and 6-month follow-up).

### Subgroup Analyses

There were no differences in the incidence of unplanned hospital visits within 12 months (primary outcome) between the CMR group and usual care in any of the predefined subgroups (age, number of prior unplanned hospital visits, number of medications, use of an automated drug-dispensing system, heart failure, chronic obstructive pulmonary disease, and type 2 diabetes) (eFigures 2-8 in Supplement 3). However, CMR plus follow-up increased the incidence of unplanned hospital visits in patients with more than 1 unplanned visit in the 12 months before inclusion (incidence rate, 3.44 visits; adjusted RR, 1.56; 95% CI, 1.20-2.05; interaction test, *P* < .001) and in patients with heart failure (incidence rate, 2.58 visits; adjusted RR, 1.36; 95% CI, 1.04-1.77; interaction test, *P* = .36) or type 2 diabetes (incidence rate, 2.56; adjusted RR, 1.34; 95% CI, 1.02-1.75; interaction test, *P* = .22) compared with usual care (eTable 5 and eFigures 1, 2, 5, and 6 in Supplement 3).

## Discussion

In this pragmatic cluster randomized crossover trial in older hospitalized patients, hospital-based CMR with and without postdischarge follow-up did not decrease the incidence of unplanned hospital visits within 12 months compared with usual care. Secondary and sensitivity analyses supported this finding, with consistent results within 30 days and 3 and 6 months. The analyses of secondary outcomes revealed an unexpected increase in the incidence of ED visits within 3, 6, and 12 months for CMR plus follow-up compared with usual care. We found no effects on medication-related admissions, visits with primary care clinicians, time to first unplanned hospital visit, mortality, or costs of hospital-based care.

Our findings are similar to those of previous RCTs on medication reviews and postdischarge interventions in hospitalized patients that provided inconclusive results on hard clinical end points.^10,28^ Medication reconciliation, medication review, and postdischarge interventions performed in isolation often seem ineffective,^28,29,30^ in contrast to similar interventions that are an integrated part of multifaceted care programs or existing care processes.^31,32,33^ Despite a mean of 2.1 recommendations to solve medication-related problems per CMR made by pharmacists, 73% of which were implemented by physicians,^22^ a process evaluation of our trial suggested that (1) the CMRs were not fully integrated in the daily workflow of the ward team, (2) the time allotted for follow-up on treatment changes was inadequate, and (3) we did not succeed in performing postdischarge interventions in collaboration with the physicians responsible for discharge and treatment follow-up.^24^ When adequate follow-up procedures are either not in place or not followed, CMRs should perhaps not be undertaken in hospitalized patients. Otherwise, the changes may increase complexity and create misunderstandings and unnecessary uncertainties for the patient, with new medication-related problems as a consequence.^34^ The results of this trial conflict with those of a 2005-2006 trial^12^ in which hospital visits were reduced by 16%. After the previous trial, medication reconciliation and medication review have been recommended in Sweden for patients who are 75 years or older and are taking 5 or more medications. Physicians and nurses have been educated on these topics through national programs and the introduction of clinical pharmacists at hospital wards^14,15^; such pursuits may have positively affected usual hospital care, providing a possible explanation for a lack of the effects that we measured in our previous trial. The counterintuitive 29% increase in ED visits among patients in the CMR plus follow-up group is more difficult to explain and should be investigated further. Patients may have responded to the intervention by becoming more vigilant in detecting any worsening of their disease symptoms, leading them to seek acute care.^35^ However, the increase in ED visits in our trial did not seem to lead to more hospital admissions or increased costs of hospital-based care, which may call into question the clinical relevance of this result.

In a recent trial in Denmark, hospital-based medication reviews that include postdischarge follow-up by pharmacists who had received a 3-day training in motivational interviewing reduced the risk of hospital readmissions in older patients.^13^ Patient empowerment and shared decision-making, as with motivational interviewing, are key factors in discharge interventions to reduce readmissions.^36^ Our process evaluation suggested that patients had a limited role in decision-making.^23^ The pharmacists were not specifically trained in shared decision-making or to empower patients through discharge counseling and follow-up telephone calls.

### Limitations

Overall, the trial was completed as planned,^22,24^ but there are some limitations to the study. First, our randomized cluster design did not allow for patient recruitment before randomization, resulting in a risk of selection bias.^37^ We mitigated this risk by screening all patients admitted to the study wards for eligibility and asking all eligible patients about participation. In the end, 356 of 2993 eligible patients (11.9%) were not included in the modified ITT analysis, indicating a low risk for a systematic between-group imbalance.

Second, there is a risk of contamination bias, despite cluster randomization. Unintended intervention components were received by 132 of the 892 control patients (14.7%) during index admission.^22^ However, only 14 control patients (1.6%) received a CMR during the index admission, indicating a low risk of contamination bias due to CMRs.

Third, the outcome measures may not fully reflect the effects of our interventions. Although 77% of the CMRs resulted in at least 1 discrepancy in the patient’s medication list or medication-related problem being resolved,^22^ the incidence of unplanned hospital visits did not decrease. Patient-reported outcomes, such as health-related quality of life, might have been more suitable for capturing effects of these treatment changes. However, subjective measurement tools, such as patient-reported outcomes, may introduce bias in trials in which ward staff and study participants cannot be blinded to treatment allocation.^38^

Finally, our interventions may not have been sufficiently targeted at a specific patient population. Although medication-related problems are common in older hospitalized patients, their subsequent use of health care may be driven by factors other than the solution of medication-related problems, such as frailty or lack of social support.^39^ We included all patients, regardless of their needs, reason for admission, condition, or medication use. Medication reviews and similar pharmacist interventions improve clinical outcomes in patients with cardiovascular disease, type 2 diabetes, chronic obstructive pulmonary disease, and cognitive impairment.^40,41,42^ In our trial, CMR was not effective in patients with previously diagnosed chronic obstructive pulmonary disease, and CMR plus follow-up even increased unplanned hospital visits in patients with heart failure and type 2 diabetes. However, neither our interventions nor our outcome measures were developed to specifically address these conditions.

## Conclusions

In this cluster randomized crossover trial of older hospitalized patients, CMRs that included postdischarge follow-up did not decrease the incidence of unplanned hospital visits compared with hospital-based reviews alone or usual care. These findings do not support the performance of hospital-based CMRs as conducted in this trial. Comprehensive medication review is a complex and multiprofessional intervention, and its success or failure is likely to be context specific. More research is needed to determine the optimal setting and timing of CMRs. Some RCTs with similar interventions aimed at improving health outcomes in older patients are currently underway.^43,44,45,46^ While we await the results of these trials, this study creates the opportunity to reflect on how to improve medication use in hospital practice and after discharge and to subject improved interventions to RCTs, with the aim of optimizing treatment outcomes and minimizing medication-related harm.

## Notes

Supplement 1. **Trial Protocol**

Supplement 2. **Statistical Analysis Plan**

Supplement 3. **eMethods 1.** Information From Trial Protocol and Statistical Analysis Plan **eMethods 2.** Sensitivity Analysis **eTable 1.** Primary Outcome Analysis for the Intention-to-Treat Population With Missing Data Imputed Under the Missing at Random Assumption **eTable 2.** Primary Outcome Analysis for the Intention-to-Treat Population With Missing Data Imputed Under the Missing Not at Random Assumption **eTable 3.** Secondary Outcomes Within 30 Days and 3, 6, and 12 Months **eTable 4.** Costs of Hospital-Based Care Within 6 and 12 Months **eTable 5.** Tests for Interaction Between Treatment Groups and Predefined Subgroups **eFigure 1.** Forest Plots for Age Subgroup Comparisons **eFigure 2.** Forest Plots for Number of Unplanned Hospital Visits Within 12 Months Before Inclusion Subgroups Comparisons **eFigure 3.** Forest Plot for Number of Prescribed Medications Upon Admission Subgroup Comparisons **eFigure 4.** Forest Plot for Use of an Automated Drug-Dispensing System in the Home Care Situation Subgroup Comparisons **eFigure 5.** Forest Plot for Previously Diagnosed Congestive Heart Failure Subgroup Comparisons **eFigure 6.** Forest Plot for Previously Diagnosed Chronic Obstructive Pulmonary Disease Subgroup Comparisons **eFigure 7.** Forest Plot for Previously Diagnosed Diabetes Mellitus Subgroup Comparisons **eReferences.**

Supplement 4. **Data Sharing Statement**
