# Incorrect Trial Registration Identifier

**DOI:** 10.1001/jamanetworkopen.2022.9745

**Published:** 2022-04-05

In the Original Investigation titled “Effects of Hospital-Based Comprehensive Medication Reviews Including Postdischarge Follow-up on Older Patients’ Use of Health Care: A Cluster Randomized Clinical Trial,”^1^ published April 30, 2021, an incorrect ClinicalTrials.gov identifier was listed for the randomized clinical trial being reported. The identifier should have been listed as NCT02999412. This article has been corrected.^1^
