# Supplementary material for: Effects of Hospital-Based Comprehensive Medication Reviews Including Postdischarge Follow-up on Older Patients’ Use of Health Care: A Cluster Randomized Clinical Trial
Source: JAMA Netw Open. 2021 Apr 30;4(4):e216303. doi: 10.1001/jamanetworkopen.2021.6303 (PMC8087955; doi:10.1001/jamanetworkopen.2021.6303)
Supplement: Supplement 1. — Trial Protocol [file jamanetwopen-e216303-s001.pdf]

Study protocol

Medication Reviews Bridging Healthcare (MedBridge): a cluster-randomised crossover trial

Swedish titel: Läkemedelsgenomgångar som överbryggande sjukvård: en klusterrandomiserad crossoverstudie

Protocol ID: MedBridge

Version: 19-02-20 (previous version: 17-02-22)

Principal investigator: Region Uppsala (RU), Sweden

Collaborators: Region Gävleborg (RG) and Region Västmanland (RV), Sweden

MedBridge research group:

Region Uppsala
Ulrika Gillespie (project leader), Uppsala University Hospital
Thomas Kempen (project coordinator), Uppsala University Hospital
Håkan Melhus, Uppsala University Hospital and Uppsala University
Maria Bertilsson, Uppsala Clinical Research Center
Elisabet Nielsen, Uppsala University

Region Gävleborg
Johanna Sulku, Gävle Hospital
Johanna Carlsson, Gävle Hospital

Region Västmanland
Karl-Johan Lindner, Västerås Hospital
Åke Tenerz, Västerås Hospital

Trial registry: Clinicaltrials.gov: NCT02999412

Official website: www.akademiska.se/medbridge

Ethical approval: Swedish Central Ethical Review Board (CEPN; registration number: Ö 21-2016) and Regional Ethical Review Board in Uppsala (EPN Uppsala; registration number: 2016-251-1)

Internal project number: 1011393 (account nr. 90100, reference nr. AS9010001)

Funding: Uppsala-Örebro Regional Research Council [grant RFR-555601, RFR-641791 and RFR-735911], Region Uppsala [grant LUL-527721, LUL-614061 and LUL-716201], Region Gävleborg [grant CFUG-658451 and CFUG-698771] and Region Västmanland [grant LTV-675921, LTV-712341, LTV-736641 and LTV-821261], the Swedish Pharmacists Association [Sveriges Farmaceuter], the Thuréus Fund for Geriatric Research [Thuréus stiftelse för främjande av geriatrisk forskning], the Geriatric Fund [Geriatriska fonden] and the Swedish Heart and Lung Association [Riksförbundet HjärtLung; grant FA 2017:38 and FA 2018:43].

Content

1. Abstract 3
2. Background 3
3. Aim 4
   3.1 Primary objectives 4
4. Methods 4
   4.1 Setting and clusters 4
   4.2 Participants 5
   4.3 Interventions 5
   4.4 Study design 7
   4.5 Randomisation 7
   4.6 Outcomes 8
   4.7 Sample size and power calculation 9
   4.8 Data collection 10
   4.9 Statistical analyses 10
5. Process evaluation and quality assurance 11
   5.1 Protocol fidelity 11
   5.2 Quantitative intervention analysis 11
   5.3 Qualitative intervention analysis 11
6. Ethical considerations 12
7. Declaration of interests 12
8. Organisation structure and publication policy 12
9. References 13
10. List of appendices 15

// 1. Abstract

Background: Mismanaged prescribing and use of medication among elderly puts major pressure on current healthcare systems. Performing a medication review, a structured critical examination of a patient's medications, during hospital stay with active follow-up into primary care could optimise treatment benefit and minimise harm. However, a lack of high quality evidence inhibits widespread implementation. This study protocol describes the rationale and design of a pragmatic cluster-randomised, crossover trial to fulfil this need for evidence.

Aim: To study the effects of hospital-initiated comprehensive medication reviews, including active follow-up, on elderly patients' healthcare utilisation compared to 1) usual care and 2) solely hospital based reviews.

Design: Multicentre, three-treatment, replicated, cluster-randomised, crossover trial.

Setting: 8 wards with a multidisciplinary team within 4 hospitals in 3 Swedish counties.

Participants: Patients aged 65 years or older, admitted to one of the study wards. Exclusion criteria: Palliative stage; residing in other than the hospital's county; medication review within the last 30 days; one-day admission.

Interventions: 1, comprehensive medication review during hospital stay; 2, same as 1 with the addition of active follow-up into primary care; 3, usual care.

Primary outcome measure: Incidence of unplanned hospital visits during a 12-month follow-up period.

Data collection and analyses: Extraction and collection from the counties' medical record system into a GCP compliant electronic data capture system. Intention-to-treat-analyses using log-linear Poisson generalized linear mixed models and frailty models. Process evaluation using qualitative and mixed-method analyses.

Relevance: This study has a high potential to show a reduction in elderly patients' morbidity, contributing to more sustainable healthcare in the long run.

2. Background

Mismanaged use of multiple medications is one of the greatest risks for healthcare related harm in the elderly and puts major pressure on current healthcare systems [1,2]. Identifying effective interventions to optimise treatment benefit and minimise harm is an international public health priority [3]. Comprehensive medication review, a structured and critical examination of an individual patient's medications in relation to the patient's conditions, aims to accomplish these goals [4]. A recently published Cochrane meta-analysis examined whether medication reviews lead to improvement in health outcomes of hospitalised adult patients compared with standard care [5]. Ten randomised controlled trials (RCTs) were eventually used for analysis. The authors reported to find no evidence that medication reviews reduce mortality or hospital admissions, although evidence that medication reviews may reduce emergency department contacts was found. A beneficial or detrimental effect on mortality or hospital admissions could be ruled out because estimates were uncertain and follow-up was short. The authors stated that there is a strong need for RCTs of high quality, with at least one year follow-up and randomised at a cluster level to minimise contamination bias [5]. In addition, a cluster randomised crossover design in which each cluster serves as its own control would increase statistical power [6].

One of the included RCTs in this meta-analysis was conducted by our research group at Uppsala University Hospital in 2005-2006 [7]. We demonstrated in this study that patients, aged 80 years or older, who received a comprehensive medication review including follow-up after hospital discharge, performed by ward based clinical pharmacists, had 16% fewer hospital revisits in the following year. The costs of hospital based care were approximately €200 lower per patient in the intervention group compared to the control group after the intervention costs had been included. The study was conducted at two internal medicine wards with 400 enrolled patients, all 80 years or older; factors that limit the generalisability and applicability of the results.

Today, ten years after our previous RCT, several counties in Sweden have employed clinical pharmacists who perform medication reviews in hospital settings. However, there are not enough resources allocated to structurally provide an intervention as extensive as the one performed in the previous study. There is thus a risk that precisely those parts of the intervention which contributed the most to the positive results, are currently not carried out in practice. For this reason it is important to identify the activities that generate the greatest benefits to the patients, something that can be done by performing a pragmatic clinical trial, specifically designed to show real-world effects of such interventions [8]. Hence we propose this pragmatic cluster-randomised, three-treatment, crossover trial to study the effects of multidisciplinary medication reviews with active follow-up after hospital discharge on elderly patients' healthcare utilisation, compared with solely hospital based reviews and usual care. This large study will generate more robust results and have high external validity, with the potential to show effects on hard outcomes such as healthcare utilisation. A similar study has, to our knowledge, not been undertaken anywhere else in the world.

3. Aim

To study the effects of hospital-initiated comprehensive medication reviews, including active follow-up, on elderly patients' healthcare utilisation compared to 1) usual care and 2) solely hospital based reviews.

3.1 Primary objectives

1. The first primary objective is to test the hypothesis that the incidence of unplanned hospital visits (admissions plus visits to the emergency department) among elderly patients during a one year follow-up is lower, if they receive a comprehensive medication review with active follow-up after discharge, than if they receive usual care.
2. The second primary objective is to test the hypothesis that a comprehensive medication review with active follow-up after discharge reduces the incidence of unplanned hospital visits more than only a medication review during hospital stay compared to usual care.

4. Methods

This study protocol has been developed in accordance with the applicable recommendations of the *SPIRIT 2013 Guidance for protocols of clinical trials*, the *Consort 2010 extension to cluster randomised trials* and the *Consort 2010 extension to randomized trials of non-pharmacologic treatment* [9-11]. A more condensed version of this study protocol has been published in the peer-reviewed journal Contemporary Clinical Trials [12].

4.1 Setting and clusters

The study will be conducted in three counties in Sweden: Västmanland, Uppsala and Gävleborg. Within these counties there will be four hospitals taking part in the study: Uppsala University Hospital and the hospitals of Västerås, Enköping and Gävle. At these hospitals clinical pharmacy, including the performance of medication reviews, is an established practice or is planned to be implemented prior to the study. The intervention components of the study will be performed by the existing workforce at two wards (internal medicine and/or geriatrics) within each hospital. The eligibility of the included wards has been based on the presence of a multidisciplinary ward staff consisting of physicians, nurses and clinical pharmacists. Next to that, the patient population within these wards consist mostly of multimorbid elderly patients who are not in the need for intensive or critical care. The clinical pharmacists have either followed a fulltime one year postgraduate programme in clinical pharmacy, in which the performance of medication reviews plays a central role, or have followed undergraduate courses in clinical pharmacy and advanced pharmacotherapy. Next to that, all pharmacists have at least six months working experience with performing medication reviews in a multidisciplinary team. To assess clinical skill performance related to medication reviews, all pharmacists participated in an objective structured clinical examination (OSCE) prior to the start of the study. All pharmacists have also participated in a training day with patient case discussions, to assure common practice across all study sites. Other members of the ward team did not receive additional training. Prior to the start

of the study, a pilot study with a minimum duration of two weeks was performed at each ward to get familiarised with the study procedures.

In total eight wards will be included, with each ward acting as a cluster. The study will be carried out as far as possible identically at the eight wards, following Standard Operating Procedures (SOPs), see appendix 1-4. However, statistical analysis will account for differences between wards, see 4.9 Statistical analyses. Randomisation will take place at cluster level, see 4.4 Study design and 4.5 Randomisation.

4.2 Participants

All patients who are eligible for inclusion according to the in- and exclusion criteria will be asked for informed consent.

Inclusion criteria

- Patients aged 65 years or older who have been admitted to one of the study wards

Exclusion criteria

- Patients who have been subject to a medication review by a clinical pharmacist within the last 30 days as stated within their electronic medical record (EMR)
- Patients residing in another than the hospital's county
- Patients in a palliative stage as stated in their EMR
- Patients admitted for only one day (maximum one overnight stay)

4.3 Interventions

Two different interventions will be compared with each other and with usual care. Detailed SOPs will serve as checklists for the clinical pharmacists' work, see appendix 2-4. The different interventions pertain to individual patients. The participant flow from a patient perspective is graphically presented in Fig. 1. The participants' schedule of enrolment including post-inclusion assessments is shown in appendix 5.

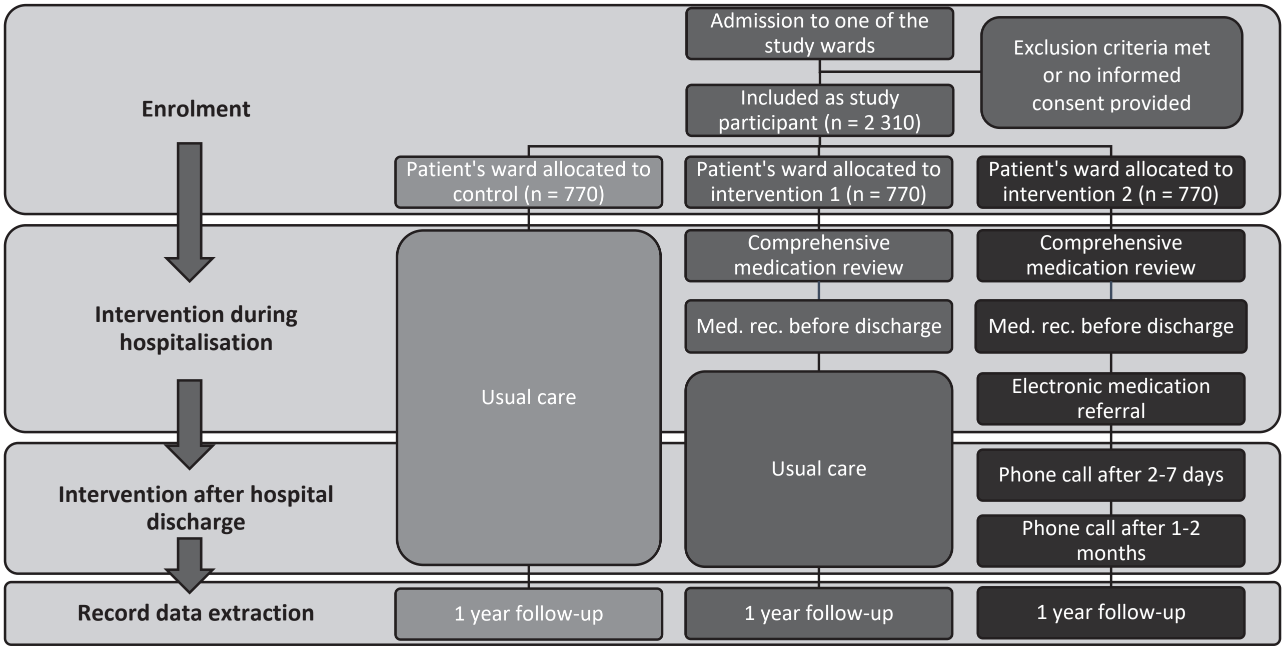

Figure 1: Participant flow from an individual patient perspective including expected number of included patients. Med. rec. = medication reconciliation

Intervention 1 (I1) 'comprehensive medication review'

- A thorough medication reconciliation within 24 hours after admission, including a patient or carer interview, by a clinical pharmacist to ensure the hospital list of medication is consistent with what

medications the patient used at home. The start of the intervention may be delayed to the pharmacist's discretion in case the patient's acute condition does not allow for an interview.

- Directly after the medication reconciliation, the clinical pharmacist performs a comprehensive medication review in collaboration with the ward physician and patient, similar to a level three clinical medication review as earlier described in the literature [4]. This includes a structured, critical examination of all of the patient's medications in relation to the patient's conditions, based on information from the patient and the EMR. The objective is to reach an agreement about the continued appropriateness and effectiveness of the treatment, optimising the impact of medications and minimising the number of medication related problems. Other issues, such as adherence, practical use of the medications (e.g. how to use an inhaler), dosage forms, adverse effects, interactions, and the patient's understanding of the condition and its treatment are considered when appropriate. The outcome of the review will be a decision about the continuation (or otherwise) of the treatment. The effects of medication changes will be monitored during the hospital stay by the physician or pharmacist, depending on the specific situation. If deemed necessary, additional contact between the physician, pharmacist and/or patient may take place during the admission to optimise medication treatment.
- Before discharge, the clinical pharmacist performs another medication reconciliation to check if the patient's medication list and prescriptions for medications to be used after hospital stay are consistent with the patient's EMR. This medication reconciliation does not necessarily involve patient contact. The pharmacist also supports the physician in making sure that the information in the EMR system is consistent with the national automated drug dispensing system, for patients enrolled in that scheme.

Intervention 2 (I2) 'comprehensive medication review with active follow-up'

The same as I1 but with the following additions:

- In case of any monitoring needs or necessary subsequent actions to be taken after hospital discharge, the clinical pharmacist and the ward physician send an electronic medication review referral to the patient's primary care physician (or any other relevant healthcare provider) upon discharge. This referral contains specific proposals and instructions regarding the patient's medication treatment for monitoring needs and necessary follow-up actions within primary care. Additional actions by the primary care physician or other clinicians are not considered a part of the intervention, but may result from this electronic referral.
- A first phone call to the patient or carer is made by the clinical pharmacist 2-7 days after discharge depending on the patient's health condition and the pharmacist's availability. All pharmacists follow a checklist to assure that the conversation addresses the same questions and topics. Within the phone call, the patient or carer will be asked to describe the current medication treatment to ensure that all information has been understood correctly. Next to that, this phone call aims to find out if any problems, concerns or questions have arisen after discharge. If necessary, the clinical pharmacist provides counselling during the phone call to solve the problems and allay concerns. Additional actions may be taken at the clinical pharmacist's discretion as a result of this phone call (e.g. sending a new medication list to the patient or carer if it is missing). Examples of situations and how to act on them will be provided and discussed with the pharmacists.
- A second phone call will be made by the clinical pharmacist approximately 30 days after hospital discharge. In case the patient has been living in a short-term stay residence after hospital discharge, this period can be extended to maximum 60 days. This phone call is primarily designed to find out how the patient is managing the medication and if any problems, concerns or questions have arisen. With decreasing treatment adherence over time, this phone call also aims to provide the patient with a motivational "boost". Subsequent contact between the clinical pharmacist and the primary care physician is not considered a mandatory part of the intervention, but may occur and will then be documented in the patient's EMR.

Control 'usual care'

- The control group will receive usual hospital care. According to Swedish legislation, usual care includes medication reconciliation upon admission. Next to that, the law requires a medication report addressing the patient's medication treatment to be given to the patient or carer upon hospital discharge and to be attached to the electronic discharge letter. This report contains a motivation for the changes in medication treatment that have been made during hospital stay, as well as the patient's updated medication list. These mandatory activities are currently carried out to various degree within the different hospitals and wards. Other activities as described in the interventions above may be carried out to a certain degree as well, but no clinical pharmacist will be involved.

4.4 Study design

A pilot study has been performed which suggested that a traditional RCT design (randomisation at patient level) was unsuitable due to practical reasons and the risk of contamination bias. For example, ward staff had to wait for informed consent and randomisation before starting the intervention and clinical pharmacists were frequently asked to review the medications of patients allocated to receive only usual care. To minimise the risk of contamination bias, it has been proposed that new RCTs on medication reviews should be randomised at a cluster level [5]. In addition, a cluster randomised crossover design in which each cluster serves as its own control increases statistical power compared to a cluster randomised parallel design [6]. Such a design produces high level evidence which forms the basis of meta-analyses and systematic reviews. The approach with two intervention groups is chosen to be able to distinguish between medication review activities during hospital stay and those after discharge. We therefore propose a cluster-randomised, three-treatment, replicated, crossover design with study periods of 8 weeks.

Crossover and randomisation will take place at ward level within each hospital, so that one ward is one cluster. This means that each ward will be allocated to one intervention (I1, I2 or control) during three consecutive periods of eight weeks. Changes over time, such as seasonal differences, and any significant changes to the cluster setting during the study's inclusion period, such as an outbreak of multi-resistant bacteria at one of the study wards, can also influence the study outcomes. To account for any of these temporal effects, the three consecutive 8-week periods will be rotated twice and randomisation of intervention sequences will be performed (see below). Contamination risk monitoring, in terms of ward personnel covering several study wards at the same time, will be performed as well. The total study duration will consist of six consecutive periods of eight weeks per ward. This means that each ward will perform each intervention for 16 active study weeks.

4.5 Randomisation

To assure that each ward performs each intervention twice, randomisation will take place at hospital level in two blocks of three periods. This within-hospital block randomisation has been chosen to spread out the different intervention periods more equally over the full study period, to account for period effects. Due to logistical, staffing reasons the method of block randomisation also prevents a particular intervention being concurrently performed on both wards within the same hospital. The randomised sequence will be generated by an independent statistician at Uppsala Clinical Research Center (UCR) using SAS© software (SAS Institute Inc., Cary, NC, USA). The computer generated codes will be held by the statistician to assure allocation concealment at the moment of randomisation. Allocation concealment will not be possible during the rest of the study. However, we regard the lack of concealment as being of minor importance due to the crossover aspect of the trial (all wards will perform each intervention twice). To minimise the possibility of selection bias within the wards, all eligible patients will be asked for informed consent to be included in the study. It will be explained to the patients that they will receive the particular intervention whether or not they provide informed consent. By this measure we aim to minimise the risk of consent bias. Any included patient that gets readmitted to one of the study wards will receive the intervention that is being performed at that particular moment. A hypothetical randomisation chart to visualise the block randomisation is shown in Fig. 2.

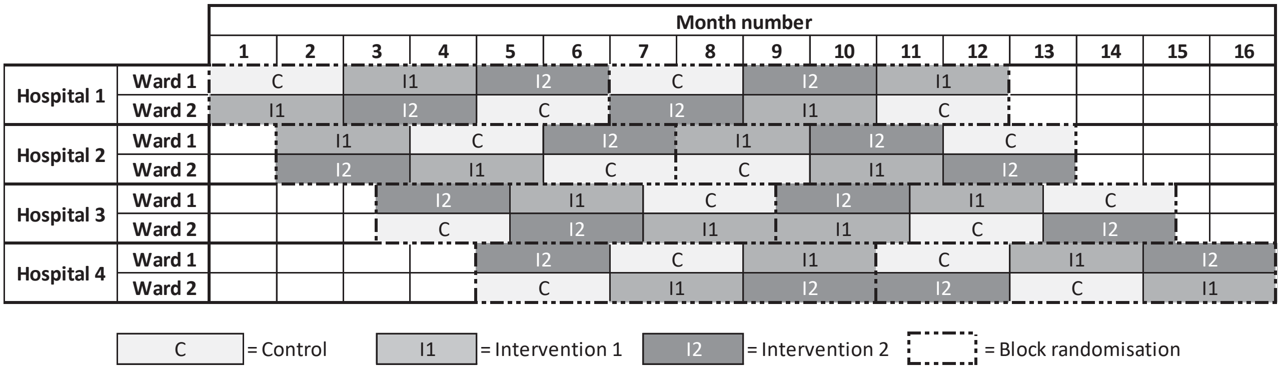

Figure 2: Chart with a hypothetical result of the block randomisation based on 6 periods of 8 weeks per ward. Each randomisation block of three periods is marked in dashed lines.

4.6 Outcomes

Primary outcome

- Incidence of unplanned hospital visits (admissions plus visits to the emergency department) during a 12-month follow-up period

An unplanned visit is defined as a visit which has not been part of the patient's treatment plan (scheduled visit), but results from an acute health problem.

Secondary outcomes

- Separate incidences of unplanned hospital admissions and emergency department visits after 30 days, 3, 6 and 12 months
- Incidences of unplanned hospital visits after 30 days, 3 and 6 months
- Separate incidences of unplanned medication related hospital admissions and unplanned primary care physician visits after 30 days, 3, 6 and 12 months
- Time from hospital discharge to first unplanned hospital visit during 12 months
- Costs of hospital based care (costs of healthcare utilisation including the costs of the intervention) after 6 and 12 months
- All-cause mortality rates after 30 days, 3, 6 and 12 months

Secondary outcomes include the primary outcome applied to the following subgroups according to baseline characteristics:

- Age: 65-74 years vs. ≥75 years
- Number of unplanned hospital visits within 12 months before admission: 0-1 vs. >1 visits
- Number of prescribed medications upon admission: <5 vs. 5-9 vs. ≥10 medications
- Using an automated drug dispensing system in the home care situation vs. no automated drug dispensing system
- Previously diagnosed diseases according to the patient's EMR: congestive heart failure (HF) vs. no HF; chronic obstructive pulmonary disease (COPD) vs. no COPD; diabetes mellitus (DM) vs. no DM

Choice of outcome measures

All primary and secondary outcomes are measurements of patients' healthcare utilisation. Unplanned hospital visits is an objective and clinically important outcome measure reflecting the patients' morbidity. Any statistical difference in the primary outcome measure is therefore considered clinically relevant. The study interventions are expected to only influence medication related hospital visits instead of all-cause hospital visits. However, due to the subjective nature of this outcome measure, we have chosen this as a secondary outcome measure. Because of a lack of available diagnostic information, it was deemed impossible to assess to what extent emergency department visits were medication related. Unplanned medication related hospital admissions was therefore chosen as an outcome measure. Next to that, unplanned primary care physician visits has been added as a relevant outcome measure for primary care practices. As this

endpoint is not of similar clinical and economical importance as hospital visits, we considered it unsuitable to combine both into one composite primary endpoint. With the time to first unplanned hospital visit we will be able to study effectiveness without the risk of contamination in terms of interventions provided to individual study participants; e.g. a study patient included as a control group patient later receiving I1 and I2 during the follow-up period. Based on data from our previous RCT and pilot study, we expect that approximately 20% of all study participants will be readmitted to one of the study wards within 12 months after discharge. Hence, there is a high risk of contamination. We have accounted for this contamination in the power calculation by lowering the expected difference in the primary outcome measure and we will perform sensitivity analyses using per-protocol (PP) analyses to investigate the effect of contamination. Nevertheless, time to first unplanned hospital visit allows for an intention-to-treat (ITT) analysis without such contamination, which justifies this additional outcome measure. We have included costs of hospital based care as a cost-effectiveness component in order to be able to value the interventions economically. Finally, all-cause mortality is added as our clinically most relevant endpoint, taking into account that our study might not be sufficiently powered to show a significant difference.

We have chosen the 30 days and 12 month follow-up periods in both the primary and secondary outcome measures to investigate the effectiveness on a short and mid-long term. These cut-off points are frequently used in clinical trials, for example in our previous RCT, which allows for between study comparisons [5,13]. Next to that, 30-day hospital readmissions is an important measure for the quality of healthcare in many countries, e.g. Sweden and the United States [13,14]. In addition, we consider three and six months as logical follow-up periods in order to be able to show trends from 30 days to 12 months follow-up.

Choice of subgroups

We have defined different subgroups based on participants' baseline characteristics in order to be able to investigate which patients benefit the most from the study interventions. According to Swedish legislation, all patients aged 75 years or older with five or more prescribed drugs in use should receive a medication reconciliation with every hospital visit and, if deemed necessary, a comprehensive medication review [15]. We therefore want to investigate whether there is a difference between patients aged 65-74 years and 75 or older in terms of effectiveness. The lower cut off point for the number of prescribed medications (<5 vs. 5-9) is also based on this legislation, whereas the other cut off point (≥10) is arbitrarily chosen based on our pilot study data: approximately 50% of the included patients used ten or more medications upon admission. Next to that, the number of previous unplanned hospital visits is considered an important predictor of our primary outcome (unplanned hospital visits). Based on our previous RCT and pilot study, the median number of previous unplanned hospital visits is expected to be one, motivating our cut off point (>1 visits). Lastly, HF, COPD and DM are seen as indications for which medication treatment plays such an essential role that any hospital admission related to these diseases are classified as preventable medication related hospital admissions in Sweden [16]. As the interventions in this study aim to optimise medication treatment, we expect to see different effectiveness in participants who have previously been diagnosed with specifically these diseases.

4.7 Sample size and power calculation

The proposed cluster-randomised crossover design will result in an approximately 1:1:1 ratio of study participants in I1, I2 and in the control group. In our previous study in which we compared a comprehensive medication review with usual care at two hospital wards, the reduction in hospital visits was 16% [7]. Due to the multicentre nature of the current study, as well as an expected 20% of the patients revisiting study wards and receiving one or more additional study interventions (possibly diluting the estimated difference between groups), the expected reduction in this study is approximately 10%. Based on our previous RCT and data from our pilot study, we expect an incidence of two hospital visits (per patient year) in the control group. This means that a 10% reduction would result in Number Needed to Treat (NNT) of five to prevent one hospital visit during the 12 months follow-up, which we consider highly clinically relevant.

Power simulations were performed using the R package clusterPower version 0.5 [17,18]. The power simulations were based on a fixed effects Poisson regression with a between cluster variance of 0.5. Analyses

at the cluster level was assumed, even though the subsequent analyses will be performed at the individual level. We used anticipated cluster sizes from the pilot study (i.e. varying cluster sizes), with eight clusters and six periods per cluster in total. The expected mean number of at-risk days per patient was 290 and we assumed seven hospital visits per 1000 patient-days in the control group. With these assumptions, 2310 study participants in total would be needed to show a 10% reduction of hospital visits between I2 and the control group with a power of approximately 83% (α=5%). The corresponding power for an expected 3% difference between I1 and I2 would be approximately 48%.

No compensation for withdrawals has been accounted for in the sample size estimation, since the primary analysis will be based on the ITT principle. We plan for an inclusion period of 12 months within each hospital which, based on the pilot study, will be sufficient to include enough patients.

4.8 Data collection

Information on patient characteristics, hospital visits and primary care physician visits will be extracted from the counties' EMR system (e.g. COSMIC®, Cambio Healthcare Systems AB, Stockholm, Sweden). The extraction will take place on a daily basis during the first hospital admission upon study inclusion and then after the pre-specified follow-up moments 30 days, 3, 6 and 12 months after hospital discharge. In case the patient is registered at a private healthcare centre without shared EMR system, the centre in question will be contacted directly to retrieve data. Data regarding mortality will be extracted from the EMR system and the national death registry. Costs of hospital based healthcare will be retrieved from the counties' costs per patient (CPP) system, and average costs of clinical pharmacist employment will be calculated to account for the intervention costs. During the inclusion phase of this study, a separate research project will focus on what the average costs of the pharmacist employment are.

Two final-year undergraduate pharmacy students will independently assess whether the hospital admissions are either unlikely to be or possibly medication related. The Assessment Tool for identifying Hospital Admissions Related to Medications (AT-HARM10) will be used for these assessments [19]. AT-HARM10 is a practical tool to identify medication related hospital admissions which is valid for use in older patients by final-year undergraduate and postgraduate pharmacy students. For an admission to be classified as medication related, consensus needs to be reached between the two students, and where this is not possible, a clinical expert will have the deciding vote. All three assessors will be blinded to study allocation and are not involved in any other part of the MedBridge trial.

Data management will follow a detailed Data Management Plan, see appendix 6. All data will be anonymised, captured in CRFs by local research assistants and saved within a protected Electronic Data Capture (EDC) system, Castor EDC©, 2016 Ciwit B.V., Amsterdam, the Netherlands. All analyses will be based on these CRF data, see appendix 7-9.

4.9 Statistical analyses

Statisticians from UCR will be responsible for the statistical analyses. Statistical analyses will primarily be performed using the ITT principle. In addition, supportive analyses will be performed using PP analyses, i.e. excluding patients and/or clusters where protocol violation had occurred, and as-treated analyses on a per-patient basis. The two primary objectives will be tested using a closed testing procedure. Patient characteristics will be presented with descriptive statistics per study group (control, I1 and I2). Rates of healthcare contacts (admissions, emergency department visits and primary care) and number of days spent in hospital will be analysed using log-linear models with Poisson variance function in the framework of Generalized Linear Mixed Models (with fixed-treatment, random-cluster and patient-within-cluster effects). The number of out-of-hospital days will be used as an offset. We will use estimated rate ratios with 95% confidence intervals (CIs) for comparison between groups. Time-to-event outcomes will be analysed using frailty models. The three groups will be compared with hazard ratios and 95%-CIs. Costs of hospital based care will be presented with descriptive statistics and the nonparametric bootstrap method will be used to compare costs and estimate confidence intervals.

No imputation for missing data will be performed. Patients that drop out will be censored at that time-point. Reasons for, and number of, drop-outs per intervention group will be reported and analysed descriptively. Tests of significance will be two-tailed and a p-value less than 0.05 will be considered significant. All statistical analyses will be performed with SAS© software (SAS Institute Inc., Cary, NC, USA) or R software (R Foundation for Statistical Computing, Vienna, Austria).

5. Process evaluation and quality assurance

Within the MedBridge study, a process evaluation consisting of continuous monitoring and several projects and sub-studies will be performed. Such a process evaluation within an RCT can provide detailed information about the different components of the interventions, for example the implementation, the mechanism of impact and the context [20]. The purpose of process evaluations is to provide a deeper understanding of the study results, which is often needed to inform policy decisions and further integration of the interventions in daily practice [21].

5.1 Protocol fidelity

Participant inclusion and informed consent rates will be updated on a daily basis. The performed intervention components (as defined by the bullet points in 4.3 Interventions) will be recorded by the clinical pharmacists as notes in the patients' EMR as currently part of their daily clinical routine. Data about the performed intervention components, to be used as protocol fidelity measures for daily monitoring and sensitivity analyses, will be extracted from the physicians' and pharmacists' notes within the EMR system during the full 12-month follow-up period.

Deviations from the protocol will be captured in the EDC system. Regular meetings will be held at each study site, to share progress on the inclusion of study participants and the performance of the study interventions among the involved researchers and clinical pharmacists. Problems and proposals for improvement will be discussed during this meeting. Beside the regular meetings, the full research group will have monthly meetings to discuss any problems that arise and to ensure that the study is progressing as planned.

5.2 Quantitative intervention analysis

As a quality measurement for I1 (comprehensive medication review) and I2 (comprehensive medication review with active follow-up), the number and type of identified medication related problems will be extracted from the EMR. For these assessments, one-third of all intervention patients will be randomly selected within each cluster and study period. Categorisation of the problems will be performed using an adjusted version of the Hepler and Strand classification of drug related problems [22]. For each problem, the proposal to solve the problem and whether this action was finally taken will be noted down based on the information within the EMR. The mean number of medication related problems per intervention and the percentage of implemented proposals will be presented.

5.3 Qualitative intervention analysis

Physicians and clinical pharmacists

A nested qualitative analysis will be performed to evaluate the implementation process of the study interventions and to promote the understanding of why the interventions within the study may or may not have been effective. Specific objectives are to identify existing barriers and facilitators to the implementation and performance of the study interventions and to investigate the attitudes and beliefs of the physicians and pharmacists towards these interventions. The qualitative analysis will use semi-structured interviews with physicians and clinical pharmacists. A similar analysis within the Pharmacists in Practice Study where researchers investigated the experience of practice staff, pharmacists and patients with pharmacist services in Australian general practice clinics, will be used as an example to draw up questions and discussion topics [23]. The interviews will also be informed by widely used frameworks such as the Structuration Model of Collaboration and the Normalization Process Theory focussing on interprofessional collaboration and the implementation of complex interventions [24,25]. We aim to involve as many different pharmacists and physicians from all study wards as possible. The interviews will be recorded and then transcribed verbatim.

The transcripts from the interviews will be analysed using thematic analysis. Finally, a mixed method analysis will be performed combining both quantitative and qualitative data.

Patients

Knowledge about the patient perspective is needed to support the understanding of the effects of comprehensive medication reviews on patients' health outcomes and to improve clinical practice. We therefore aim to explore older patients' experiences with, and views on, hospital-initiated comprehensive medication reviews and follow-up telephone calls by ward-based clinical pharmacists within the MedBridge trial. In-depth semi-structured interviews will be conducted with study participants (and/or their carers) who received one of the two active study interventions (I1 or I2). Purposive sampling will be used to be able to identify themes which cut across a variety of patients [26]. In this approach, patients will be sampled to ensure coverage across hospital wards and heterogeneity in terms of age, gender, intervention (I1 or I2), clinical pharmacist involved, and the number (high or low) of medication related problems identified during the comprehensive medication review. Sampling will be continued to the point of data saturation, defined as the point when no new additional data are found that develop a conceptual category or theme [27]. In our approach, 12 initial interviews will be conducted and analysed, to identify key, recurring themes. After 12 interviews, three consecutive interviews with no new themes emerging are needed to reach the point of data saturation [27]. Discussion topics include communication, information, decision-making, and effects on the patient. Interviews will take place after discharge, and will be audio-recorded, transcribed verbatim, and thematically analysed using a framework approach as proposed by Ritchie and Spencer [26].

6. Ethical considerations

This study has received ethical approval from the Swedish Central Ethical Review Board (CEPN; registration number: Ö 21-2016) and additional ethical approval from the Regional Ethical Review Board in Uppsala for the patient interviews (EPN Uppsala; registration number: 2016-251-1). Due to the nature of the study design, written informed consent is not needed prior to the start of the intervention, but only for collection of individual patient data. All participants and their data will be handled according to the ethical principles as stated in the WMA Declaration of Helsinki [28]. Next to that, this study complies with all applicable recommendations of the ICH-GCP standards [29]. With this trial we aim to gain essential knowledge about which medication review activities generate the most benefit on clinically relevant outcomes and when during the care process these effects are the greatest. We will only include study sites where clinical pharmacists, who perform medication reviews on a daily basis, are already integrated in the ward team. The two interventions within this study consist of activities which are in line with current Swedish guidelines regarding medication reviews, with the addition of extra patient consultation and follow-up communication with patients and primary care physicians. The control group will receive usual hospital care. As there are currently not enough pharmacist resources to provide medication reviews to all elderly patients, we consider the use of a control group receiving usual care to be justifiable. Next to that, the performance of medication reviews within a hospital setting is currently (still) not common practice in most hospitals in Sweden. No patient will be at increased risk of harm in relation to standard hospital care.

7. Declaration of interests

All research group members declare to have no conflict of interest.

8. Organisation structure and publication policy

Our research group consists of representatives from Region Uppsala, Region Gävleborg and Region Västmanland, which are all part of the Uppsala-Örebro healthcare region. Within this region there is a long history of strong collaboration when it comes to innovations in healthcare. Specifically, the current medication review activities of the three different project partners result from close collaboration between healthcare professionals within this region. The MedBridge research group decides on matters regarding authorship and contribution. The research group will follow the recommendations of the International Committee of Medical Journal Editors (ICMJE) [30]. A reference group has been formed to advice the research

group on study related matters. The reference group consists of experienced clinicians, managers and researchers from Sweden as well as Norway, see the Study Contact List (appendix 10). The reference group receives important documents, such as the updated version of the study protocol, and all minutes from research group meetings. The group members are invited to give advice to the research group and will be actively approached in case of important decisions.

9. References

1) The Swedish National Board of Health and Welfare. Current developments in care of the elderly in Sweden. Stockholm: Socialstyrelsen, 2007. Article nr: 2007-131-40.
2) Chiatti C, Bustacchini S, Furneri G, Mantovani L, Cristiani M, Misuraca C, et al. The economic burden of inappropriate drug prescribing, lack of adherence and compliance, adverse drug events in older people: a systematic review. Drug Saf. 2012 Jan;35 Suppl 1:73-87.
3) The WHO Research Priority Setting Working Group. Global Priorities for Research in Patient Safety (first edition): World Health Organization, 2008.
4) Blekinsopp A, Bond C, Raynor DK. Medication reviews. Br J Clin Pharmacol 2012; 74: 573–80.
5) Christensen M, Lundh A. Medication review in hospitalised patients to reduce morbidity and mortality. Cochrane Database of Systematic Reviews 2016, Issue 2. Art. No.: CD008986.
6) Rietbergen C, Moerbeek M. 2011. The design of cluster randomized crossover trials. J. Educ. Behav. Stat. 36:472–90.
7) Gillespie U, Alassaad A, Henrohn D, Garmo H, Hammarlund-Udenaes M, Toss H, Melhus H, Mörlin C. A comprehensive pharmacist intervention to Reduce morbidity in patient 80 Years or Older: a randomized controlled trial. Arch Intern Med. 2009 May 11; 169 (9): 894-900.
8) Ford I, Norrie J. Pragmatic Trials. N Engl J Med. 2016 Aug 4;375(5):454-63. doi: 10.1056/NEJMra1510059.
9) Chan AW, Tetzlaff JM, Gøtzsche PC, Altman DG, Mann H, Berlin JA, et al. SPIRIT 2013 explanation and elaboration: guidance for protocols of clinical trials. BMJ. 2013 Jan 8;346:e7586.
10) Campbell MK, Piaggio G, Elbourne DR, Altman DG; CONSORT Group. Consort 2010 statement: extension to cluster randomised trials. BMJ. 2012 Sep 4;345:e5661.
11) Boutron I, Moher D, Altman DG, Schulz KF, Ravaud P; CONSORT Group. Extending the CONSORT statement to randomized trials of nonpharmacologic treatment: explanation and elaboration. Ann Intern Med. 2008 Feb 19;148(4):295-309.
12) Kempen TGH, Bertilsson M, Lindner KJ, Sulku J, Nielsen EI, Högberg A, et al. Medication Reviews Bridging Healthcare (MedBridge): Study protocol for a pragmatic cluster-randomised crossover trial. Contemp Clin Trials. 2017 Oct;61:126-132. doi: 10.1016/j.cct.2017.07.019. Epub 2017 Jul 21
13) Renaudin P, Boyer L, Esteve MA, Bertault-Peres P, Auquier P, Honore S. Do pharmacist-led medication reviews in hospitals help reduce hospital readmissions? A systematic review and meta-analysis. Br J Clin Pharmacol. 2016 Aug 11.
14) Leppin AL, Gionfriddo MR, Kessler M, Brito JP, Mair FS, Gallacher K, et al. Preventing 30-day hospital readmissions: a systematic review and meta-analysis of randomized trials. JAMA Intern Med. 2014 Jul;174(7):1095-107.
15) The Swedish National Board of Health and Welfare. Changes in regulations and general guidelines (SOSFS 2000:1) of medication use in healthcare [in Swedish]. SOSFS 2012:9 (M). Västerås: Socialstyrelsen, 2012. ISSN 0346-6000; Artikelnr.2012-6-43.
16) The Swedish National Board of Health and Welfare. Development of indicators for avoidable hospital care and unplanned readmissions [in Swedish]. Stockholm: Socialstyrelsen, 2014. Article nr: 2014-2-12.
17) Reich NG, Obeng D. clusterPower: Power calculations for cluster-randomized and cluster-randomized crossover trials. R package version 0.5. CRAN.Rproject.org/package=clusterPower (2013).
18) Reich NG, Myers JA, Obeng D, Milstone AM, Perl TM. Empirical power and sample size calculations for cluster-randomized and cluster-randomized crossover studies. PLoS One 2012, 7(4):e355-64.
19) Kempen TGH, Hedström M, Olsson H, Johansson A, Ottosson S, Al-Sammak Y, et al. Assessment tool for hospital admissions related to medications: development and validation in older patients. Int J Clin Pharm. 2019 Feb;41(1):198-206. doi: 10.1007/s11096-018-0768-8. Epub 2018 Dec 26.
20) Oakley A, Strange V, Bonell C, Allen E, Stephenson J, RIPPLE Study Team. Process evaluation in randomised controlled trials of complex interventions. BMJ. 2006 Feb 18;332(7538):413–6.
21) Moore GF, Audrey S, Barker M, Bond L, Bonell C, Hardeman W, et al. Process evaluation of complex interventions: Medical Research Council guidance. BMJ. 2015 Mar 19;350:h1258.
22) Hepler CD, Strand LM. Opportunities and responsibilities in pharmaceutical care. Am J Hosp Pharm 1990;47(3):533-43.

23) Tan ECK, Stewart K, Elliott RA, George J. Stakeholder experiences with general practice pharmacist services: a qualitative study. BMJ Open. 2013 Sep 1;3(9):e003214.
24) May, C., Rapley, T., Mair, F.S., Treweek, S., Murray, E., Ballini, L., Macfarlane, A. Girling, M. and Finch, T.L. (2015) Normalization Process Theory On-line Users' Manual, Toolkit and NoMAD instrument. Available from http://www.normalizationprocess.org.
25) D'Amour D, Goulet L, Labadie JF, Martín-Rodriguez LS, Pineault R. A model and typology of collaboration between professionals in healthcare organizations. BMC Health Serv Res. 2008 Sep 21;8:188.
26) Ritchie J, Lewis J. Qualitative research practice: a guide for social science students and researchers. Sage Publications; 2003. 336 p.
27) Francis JJ, Johnston M, Robertson C, Glidewell L, Entwistle V, Eccles MP, et al. What is an adequate sample size? Operationalising data saturation for theory-based interview studies. Psychol Heal. 2010;25:1229-45.
28) World Medical Association. World Medical Association Declaration of Helsinki: ethical principles for medical research involving human subjects. JAMA. 2013 Nov 27;310(20):2191-4.
29) ICH. ICH harmonised tripartite guideline for good clinical practice E6(R1), International Conference on Harmonisation of Technical Requirements for Registration of Pharmaceuticals for Human Use [Internet]. ICH; 1996. Current Step 4 version; dated 10 June 1996 [Cited 08-09-2016]. Available from: www.ich.org.
30) ICMJE. Recommendations for the Conduct, Reporting, Editing, and Publication of Scholarly work in Medical Journals [Internet]. ICMJE; 2015 [Cited 08-09-2016]. Available from: www.icmje.org.

10. List of appendices

Nr.	Item
1	Standard Operating Procedure – Clinical Pharmacists
2	Standard Operating Procedure – Coordinator Uppsala/Enköping
3	Standard Operating Procedure – Coordinator Gävle
4	Standard Operating Procedure – Coordinator Västerås
5	Schedule of Enrolment, Interventions and Assessments
6	MedBridge Data Management Plan
7	Electronic Case Report Form: General part
8	Electronic Case Report Form: Reports
9	Electronic Case Report Form: Variable lists and field options
10	MedBridge Study contact list
