# Supplementary material for: Effects of Hospital-Based Comprehensive Medication Reviews Including Postdischarge Follow-up on Older Patients’ Use of Health Care: A Cluster Randomized Clinical Trial
Source: JAMA Netw Open. 2021 Apr 30;4(4):e216303. doi: 10.1001/jamanetworkopen.2021.6303 (PMC8087955; doi:10.1001/jamanetworkopen.2021.6303)
Supplement: Supplement 2. — Statistical Analysis Plan [file jamanetwopen-e216303-s002.pdf]

Statistical Analysis Plan

**Study Code:**	MedBridge
**Study Title:**	Medication Reviews Bridging Healthcare (MedBridge): a cluster-randomised crossover trial
**Based on protocol version and date:**	2019-02-20 (previous version: 17-02-22)
**Biostatistician:**	Nermin Hadziosmanovic
**Clinical Project Leader:**	Ulrika Gillespie
**Project Coordinator:**	Thomas Kempen
**Principal Investigator:**	Uppsala University Hospital (Akademiska sjukhuset), Sweden
**Trial registry:**	ClinicalTrials.gov: NCT02999412

Reviewed and approved by Biostatistician (UCR);

Name: **Signature:** **Date:**

Approved by Principal Investigator's Representative;

Name: **Signature:** **Date:**

SAP version: Final

Table of Contents

1. Introduction 3
2. Abbreviations 3
3. Study Objectives and Endpoints 5
3.1. Objectives 5
3.1.1. Primary Objective 5
3.2. Endpoints 5
3.2.1. Primary Endpoint 5
3.2.2. Secondary Endpoints 5
4. Study design 6
5. Definition of Analysis Populations 7
6. Description of statistical analysis 7
6.1. General 7
6.2. Patient and Cluster Characteristics 7
6.3. Primary analyses 8
6.4. Secondary analyses 8
6.5. Subgroup analyses 9
6.6. Overdispersion 10
6.7. Handling of Missing Data 10
7. Determination of sample size 10
8. Changes in the Planned Analysis 11
9. Description of Derived Variables 12
10. Description of Output 14
11. Statistical software 14
12. References 14

1. Introduction

The aim of this statistical analysis plan (SAP) is to describe details of planned statistical analyses and presentation of the study data described to be performed by the UCR (Uppsala Clinical Research Center) Statistics Section. UCR will perform all analyses of primary and secondary outcome measures and pre-specified subgroups. Data on costs of hospital-based care became available in mid-2020 and have been added to the previous agreement between the principal investigator and UCR. This second final version of the SAP has been updated to include analysis of costs of hospital-based care.
The results will be presented according to the output specification (see Appendix 1 Output Shells). Study biostatistician is responsible for writing the plan with necessary input from other members of the study team. A biostatistician not otherwise involved in the study together with the coordinating investigator will approve the final version.

The SAP is based on the study protocol:
Protocol ID: MedBridge Version: 19-02-20

Analysis will start once all data for the last included patient (12-month follow-up) have been obtained, the database has been cleaned and locked, and the SAP has been finalized.

2. Abbreviations

ADD	Automated drug dispensing system
BMI	Body Mass Index
C	Control group (usual care)
CI	Confidence interval
COPD	Chronic Obstructive Pulmonary Disease
DM	Diabetes Mellitus
DMP	Data Management Plan
eCRF	Electronic Case Report Form
EDC	Electronic Data Capture system (Castor EDC in this study)
FU	Follow-up
HF	Congestive Heart Failure
ITT	Intention to Treat
mITT	Modified Intention to Treat
I1	Intervention 1 (medication review during hospital stay)
I2	Intervention 2 (medication review during hospital stay with active follow-up after discharge)
RR	Incidence Rate Ratio
RG	Region Gävleborg
RU	Region Uppsala
RV	Region Västmanland
SAP	Statistical Analysis Plan
SD	Standard Deviation
SEK	Swedish Krona
SOP	Standard Operating Procedures
UAS	Uppsala University Hospital
UCR	Uppsala Clinical Research center

3. Study Objectives and Endpoints

3.1. Objectives

3.1.1. Primary Objective

a) The first primary objective is to test the hypothesis that the incidence of unplanned hospital visits (admissions plus visits to the emergency department) among elderly patients during a one-year follow-up is lower, if they receive a comprehensive medication review with active follow-up after discharge, than if they receive usual care.
b) The second primary objective is to test the hypothesis that a comprehensive medication review with active follow-up after discharge reduces the incidence of unplanned hospital visits more than only a medication review during hospital stay compared to usual care.

3.2. Endpoints

3.2.1. Primary Endpoint

The primary endpoint is incidence of unplanned hospital visits (admissions plus visits to the emergency department) during a 12-month follow-up period. An unplanned visit is defined as a visit which has not been part of the patient's treatment plan (scheduled visit), but results from an acute health problem.

3.2.2. Secondary Endpoints

- Separate incidences of unplanned hospital admissions and emergency department visits after 30 days, 3, 6 and 12 months
- Incidences of unplanned hospital visits after 30 days, 3 and 6 months
- Separate incidences of *unplanned medication related hospital admissions* and *unplanned primary care physician visits* after 30 days, 3, 6 and 12 months
- Time from hospital discharge to first unplanned hospital visit during 12 months
- Costs of hospital-based care (total costs of all-cause unplanned hospital visits plus costs for the intervention) after 6 and 12 months
- All-cause mortality rates after 30 days, 3, 6 and 12 months

Secondary outcomes include the primary outcome applied to the following subgroups according to baseline characteristics:

- Age: 65-74 years vs. ≥75 years
- Number of unplanned hospital visits within 12 months before admission: 0-1 vs. >1 visits
- Number of prescribed medications upon admission: <5 vs. 5-9 vs. ≥10 medications
- Using an automated drug dispensing system in the home care situation vs. no automated drug dispensing system

- Previously diagnosed diseases according to the patient's electronic health record: congestive heart failure (HF) vs. no HF; chronic obstructive pulmonary disease (COPD) vs. no COPD; diabetes mellitus (DM) vs. no DM

4. Study design

This is a cluster-randomised, three-treatment, replicated, crossover design with study periods of 8 weeks.[4,5]
Crossover and randomisation took place at ward level within each hospital, so that one ward is one cluster. This means that each ward was allocated to one intervention (I1, I2 or control) during three consecutive periods of eight weeks. Changes over time, such as seasonal differences, and any significant changes to the cluster setting during the study's inclusion period, such as an outbreak of multi-resistant bacteria at one of the study wards, can also influence the study outcomes. To account for any of these temporal effects, the three consecutive 8-week periods were rotated twice, and randomisation of intervention sequences was performed (see Fig. 1). Contamination risk monitoring, in terms of ward personnel covering several study wards at the same time, was performed as well. The total study duration consists of six consecutive periods of eight weeks per cluster. This means that each cluster performed each intervention for 16 active study weeks. To assure that each cluster performed each intervention twice, randomisation took place at hospital level in two blocks of three periods. This within-hospital block randomisation was chosen to spread out the different study periods more equally over the full study period, to account for period effects. Due to logistical, staffing reasons the method of block randomisation also prevents a particular intervention being concurrently performed on both wards within the same hospital. Allocation concealment was not possible during the rest of the study. However, we regard the lack of concealment as being of minor importance due to the crossover aspect of the trial (all clusters will perform each intervention twice). To minimise the possibility of selection bias within the clusters, all eligible patients were asked for informed consent to be included in the study. It was explained to the patients that they would receive the particular intervention whether or not they provided informed consent. By this measure we aimed to minimise the risk of consent bias. Any included patient that was readmitted to one of the study wards received the intervention that was being performed at that particular moment. A hypothetical randomisation chart to visualise the block randomisation is shown in Fig. 1.

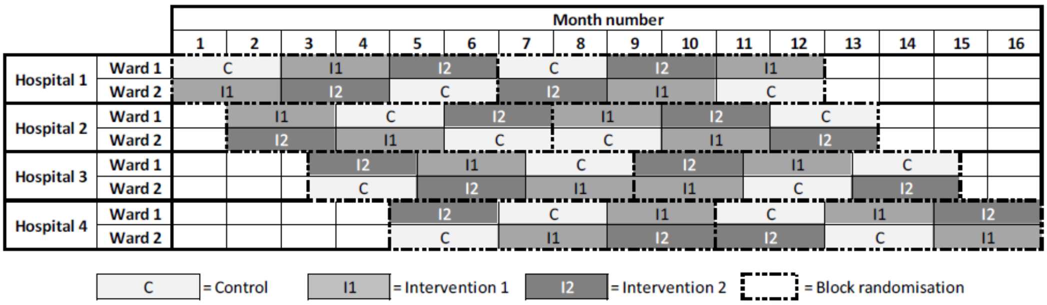

Figure 1 Chart with a hypothetical result of the block randomisation based on 6 periods of 8 weeks per ward. Each randomisation block of three periods is marked in dashed lines.

5. Definition of Analysis Populations

Dropouts	Patients that where eligible for the study (according to the in- and exclusion criteria) but did not provide informed consent and were not included in the study.
Withdrawals	Patients that fulfil inclusion criteria and have signed informed consent but withdrew after inclusion (before data were collected)
Intention to treat (ITT)	All patients that were eligible for the study (including patients that did not provide informed consent but who were eligible). All participants will be analysed according to the intervention group (C, I1 or I2) to which the cluster (ward) was allocated upon admission (index admission). Withdrawals will be included.
Modified Intention to treat (mITT)	All patients that fulfil inclusion criteria and have signed informed consent. All participants with a recorded outcome will be included in the analysis and will be analysed according to the intervention group to which the cluster was allocation upon inclusion (index admission)

The ITT population will only be used as a supportive analysis for the primary endpoint. The mITT population will be used in primary endpoint analyses and all other analyses.

6. Description of statistical analysis

6.1. General

All analyses will account for the cluster-randomized crossover design to ensure correct type I error rates and confidence intervals (CIs). A significance level of 0.05 (two-tailed) will be used for all statistical comparisons.

6.2. Patient and Cluster Characteristics

A table that summarizes number of patients by intervention group (C, I1 and I2) in total and by cluster, will be constructed. Number of patients in each intervention group will be presented together with number (proportion) and reason of drop-outs among intervention groups.

All endpoints will be summarized by intervention group, at both subject, period and cluster level (ward) using descriptive statistics.
Patients and cluster characteristics will be presented by intervention group (C, I1 and I2) in a table, but no formal statistical hypothesis testing will be performed. Continuous variables will be summarized by number of observations (n), mean, standard deviation (SD), median, minimum (min) and maximum (max). Categorical variables will be summarized in frequency tables (presenting frequencies and proportions).

6.3. Primary analyses

The differences in the incidences of unplanned hospital visits during a 12-month follow-up period (admissions plus visits to the emergency department) between intervention groups (C, I1, I2) will be compared using log-linear models with Poisson variance function in the framework of Generalized Linear Mixed Models with adjustment for the cluster effect (ward) and period effect. Number of unplanned hospital visits within 12 months before index admission (continuous variable) will be included as a patient level covariate. Number of unplanned hospital visits before admission may be categorized (0,1,2,3, 4 and >4 admissions). The number of out-of-hospital-days (log-transformed) will be used as offset.
Pairwise comparisons will be performed:
I2 vs C
I2 vs I1
I1 vs C

Results will be presented as p-value, estimated rate ratio (RR) and 95% CI for comparison between groups. Tukey´s adjusted p-values and 95% CI may be presented for pairwise comparisons to prevent multiple testing problem.
SAS procedure GLIMMIX will be used [6,7].

6.4. Secondary analyses

Incidences of

- Unplanned hospital admissions after 30 days, 3, 6 and 12 months
- Emergency department visits after 30 days, 3, 6 and 12 months,
- Unplanned hospital visits after 30 days, 3 and 6 months
- Unplanned <u>medication related hospital</u> admissions after 30 days, 3, 6 and 12 months
- Unplanned <u>primary care physician</u> visits after 30 days, 3, 6 and 12 months

will be performed using the same statistical methods as for the primary objective (other definition of the outcome variable and offset, see section 9). All above analyses will be adjusted for number of unplanned hospital visits within 12 months before index admission.

Time to event analyses:

- Time to first unplanned visit during 12 months
- All-cause mortality after 30 days
- All-cause mortality after 3 months
- All-cause mortality after 6 months
- All-cause mortality after 12 months

will be analysed with nested frailty models including gamma distributed random effect. Period will be implemented as fixed and cluster (ward) as random effect. Kaplan Meier curves will be constructed for each outcome definition. Number of unplanned hospital visits within 12 months before index admission will be included as a patient level covariate. Results will be presented as p-value, Hazard Ratio (HR) and 95% CI for comparison between groups. Pairwise comparisons may be used. EFRON method will be used for handling of ties in failure times.[8]
SAS procedure PHREG will be used.

Costs of hospital based care (total costs of all-cause unplanned hospital visits plus costs for the intervention) after

i) 6 months
ii) 12 months

will be presented as mean (SD) and median values with interquartile range (IQR; 25th–75th percentiles) by each intervention group (C, I1, I2). Average costs for each intervention (I1 and I2) will be based on time measurements of clinical pharmacists' work during different study periods and an average clinical pharmacist hourly wage derived from wage statistics of the Swedish Pharmacists Association for 2018.[9] Mean intervention costs will be added for each patient (I1 and I2). Non-parametric bootstrap method [10] will be used to compare mean costs and to estimate confidence intervals.

6.5. Subgroup analyses

The differences in the incidences of *unplanned hospital* visits *during a 12-month follow-up period (admissions plus visits to the emergency department)* between intervention groups (C, I1, I2) will be analyses in following subgroups according to baseline characteristics:

- Age (65-74 vs. ≥ 75 years)
- *Number of unplanned hospital visits within 12 months before admission: (0–1 vs.> 1 visits)
- Number of prescribed medications upon admission (< 5 vs. 5–9 vs.≥ 10 medications)
- Using an automated drug dispensing system in the home care situation vs. no automated drug dispensing system
- Previously diagnosed diseases according to the patient's medical record:
  i) congestive heart failure (HF) vs. no HF
  ii) chronic obstructive pulmonary disease (COPD) vs. no COPD
  iii) diabetes mellitus (DM) vs.no DM.

We will identify possible groups that respond better or worse to the intervention. A test for interaction, using multivariable models, to evaluate for statistically significant subgroup differences will therefore be used. Interactions will be explored graphically. We will present the outcome means for the interaction between intervention group and each subgroup. Subgroup analyses will then be performed, and results will be presented as p-value, estimated rate ratio (RR) and 95% CI for comparison between treatments by each subgroup.

NOTE: *This subgroup analysis will not be adjusted for number of unplanned hospital visits within 12 months before index admission.

6.6. Overdispersion

A restriction in the use of Poisson models is that the model assumes that the variance is equal to the mean. We can therefore often observe overdispersion in such models, i.e. the actual observed variation is too large to fit the theoretical model. An indication of overdispersion is that Persons ratio (values of Gener. Chi-Square /DF) is (much) larger than 1, which may be the case in our models. Running overdispersed Poisson models will generate understated standard errors and can lead to erroneous conclusions. We will therefore take that into account by adding a multiplicative overdispersion parameter to the variance function in all our GLIMMIX models.

6.7. Handling of Missing Data

Definition of ITT population requires that dropouts are included. Primary outcome variable (number of unplanned visits) will be imputed for patients that drop out and withdrawals, about 10% of the total number of included patients. All necessary information (period, cluster and planned intervention groups) for drop-outs is available. Multiple imputation (MI) method will be used to deal with missing data in the primary outcome variable. We will use SAS function PROC MI and generate ten imputed datasets (to ensure that our effect estimates will not overlay inaccurate due to Monte Carlo variability).[11] The results for each imputation will be combined using SAS function PROC MIANALYZE.

We will perform two sensitivity analyses:

1. According to MAR (missing at random) assumption.
   We assume that missingness is conditional on study period, intervention group and cluster and then perform the sensitivity analyses.
2. According to MNAR (missing not at random) assumption
   We will use an adjust option where imputed values for observations in the intervention group (I2) are adjusted using the shift parameter. (SHIFT option adds a constant to the imputed value). In this way, the expected mwan of the intervention group I2 will be higher than that of the corresponding of the observed I2 values. We will investigate which shift parameter reverses the study conclusion.

Regarding sensitivity analyses: We will not be able to adjust for the number of out-of-hospital-days and the number of unplanned hospital visits within 12 months before index admission because we don't have these variables for dropouts.

7. Determination of sample size

The proposed cluster-randomised crossover design will result in an approximately 1:1:1 ratio of study participants in I1, I2 and in the control group. In our previous study in which we compared a comprehensive medication review with usual care at two hospital wards, the reduction in hospital visits was 16% [1]. Due to the multicentre nature of the current study, as well as an expected 20% of the patients revisiting study wards and receiving one or more additional study interventions (possibly diluting the estimated difference between groups), the expected reduction in this study is approximately 10%. Based on our previous RCT and data from our pilot study, we expect an incidence of two hospital visits (per patient year) in

the control group. This means that a 10% reduction would result in Number Needed to Treat (NNT) of five to prevent one hospital visit during the 12 months follow-up, which we consider highly clinically relevant.
Power simulations were performed using the R package clusterPower version 0.5 [2,3]. The power simulations were based on a fixed effects Poisson regression with a between cluster variance of 0.5. Analyses at the cluster level was assumed, even though the subsequent analyses will be performed at the individual level. We used anticipated cluster sizes from the pilot study (i.e. varying cluster sizes), with eight clusters and six periods per cluster in total. The expected mean number of at-risk days per patient was 290 and we assumed seven hospital visits per 1000 patient-days in the control group. With these assumptions, 2310 study participants in total would be needed to show a 10% reduction of hospital visits between I2 and the control group with a power of approximately 83% (α=5%). The corresponding power for an expected 3% difference between I1 and I2 would be approximately 48%.
No compensation for withdrawals has been accounted for in the sample size estimation, since the primary analysis will be based on the mITT population.

8. Changes in the Planned Analysis

1. The following planned analysis is deleted because it was not feasible to define the PP-population:

"In addition, supportive analyses will be performed using PP analyses, i.e. excluding patients and/or clusters where protocol violations have occurred, and as-treated analyses on a per-patient basis."

Instead, we will use the ITT population when performing supportive analyses for the primary endpoint and the mITT population for the primary and secondary analysis.

2. The following planned analysis described in study protocol section 3.9 will be changed:

"In order to control the overall type I error, the two primary objectives will be tested in a hierarchical fashion starting with I2 versus control. If the p-value for this comparison is below 0.05, the test for I1 versus control will be performed at the 0.05 level. If the p-value for the first comparison is above 0.05, formal testing for the second comparison will not be undertaken"

All pairwise comparisons will be performed instead. Tukey´s method will be used to prevent multiple testing problem. Both adjusted and unadjusted CI will be presented.

9. Description of Derived Variables

All-cause mortality	Defined as time from inclusion until death from any cause. Status will be defined as 1=dead, 0=alive or censored.<br>If Avliden_datum ne . then dead=1; If Avliden_datum =. then dead=0
Death days	Time to death. Censored at 365 days.<br>Death_days = Avliden_datum minus Indexdatum<br>If Death_days=. then Death_days=365
All-cause mortality after 30 days	Defined as time from inclusion until death from any cause within 30 days. Status will be defined as 1=dead, 0=alive or censored.<br>If Death_days <31, then "1", otherwise "0"
Death days after 30 days	Death days censored at 30 days<br>Death_days_1M= Death_days<br>If Death_days>30 then Death_days_1M=30
All-cause mortality after 3 months	Defined as time from inclusion until death from any cause within 3 months. Status will be defined as 1=dead, 0=alive or censored.<br>If Death_days <91, then "1", otherwise "0"
Death days after 3 months	Death days censored at 3 months<br>Death_days_3M= Death_days<br>If Death_days>90 then Death_days_3M=90
All-cause mortality after 6 months	Defined as time from inclusion until death from any cause within 6 months. Status will be defined as 1=dead, 0=alive or censored.<br>If Death_days <181, then "1", otherwise "0"
Death days after 6 months	Death days censored at 6 months<br>Death_days_6M= Death_days<br>If Death_days>180 then Death_days_6M=180
Length of stay	If typ="Sjukhusvistelse" and indexvistelse=0 then days= kontakt_slut_date- kontakt_start_date +1<br>If several hospital visits, summarize all "days" within the patient.
Number of days at risk	365 minus sum of Length_of _stay (by patient).<br>This variable will be used as offset in GLIMMIX
Unplanned hospital Admissions	Unplanned Hospital admissions and (no index)<br>If typ="Sjukhusvistelse" and oplanerad=1 and indexvistelse=0 then UHA_12=1<br>4 variables will be created, UHA_12,UHA_6,

	UHA_3 and UHA_1 which corresponds to UHA after 1,3,6 and 12 months.
Emergency department visits	If typ="Akutbesök" then ED_visit_12=1<br>4 variables will be created, ED_visit _12, ED_visit _6, ED_visit _3 and ED_visit _1 which corresponds to number of emergency department visits after 1,3,6 and 12 months, respectively.
Unplanned medication related hospital admissions	if UHA_12=1 and MRA=1 then MRA_visis_12=1<br>4 variables will be created, MRA _visit _12, MRA _visit_6, MRA _visit _3 and MRA _visit _1 which corresponds to number of medication related hospital admissions after 1,3,6 and 12 months, respectively.
Unplanned primary care physician visits	if typ="Primärvårdsbesök" and kategori="Läkare/läkarstudent" then GP_visit_12=1<br>4 variables will be created, GP _visit _12, GP _visit _6, GP _visit _3 and GP _visit _1 which corresponds to number of primary care physician visits after 1,3,6 and 12 months, respectively
Primary outcome	Unplanned hospital admissions plus visits to the emergency department after 12 months.<br><br>Primary_outcome=ED_visit_12=1 plus UHA_12=1<br><br>3 secondary outcome variables will be created, Secondary_outcome _6, Secondary _outcome _3 and Secondary _outcome _1 which corresponds to number of unplanned hospital visits after 1,3 and 6 and 12 months, respectively.
Time to first unplanned visit	if first primary outcome=1 then time_to_first_visit= kontakt_start_date minus Indexdatum (within 12 months)<br>Patients with no unplanned visits will be censored at 365 days.
Cost_6M	Sum of cost of hospital-based care (in SEK) within 6 months
Cost_12M	Sum of cost of hospital-based care (in SEK) within 12 months

10. Description of Output

See Appendix 1: MedBridge SAP Appendix 1 (OS)

11. Statistical software

SAS version 9.4 or later and R (version 3.2.2) will be used.

12. References

[1] Gillespie U, Alassaad A, Henrohn D, Garmo H, Hammarlund-Udenaes M, Toss H, Melhus H, Mörlin C. A comprehensive pharmacist intervention to Reduce morbidity in patient 80 Years or Older: a randomized controlled trial. Arch Intern Med. 2009 May 11; 169 (9): 894-900.

[2] Reich NG, Obeng D. clusterPower: Power calculations for cluster-randomized and cluster-randomized crossover trials. R package version 0.5. CRAN.Rproject.org/package=clusterPower (2013).

[3] Reich NG, Myers JA, Obeng D, Milstone AM, Perl TM. Empirical power and sample size calculations for cluster-randomized and cluster-randomized crossover studies. PLoS One 2012, 7(4):e355-64.

[4] Jean-JacquesParienti, Oliver Kuss. Cluster-crossover design: A method for limiting clusters level effect in community-intervention studies. June 2007 Contemporary Clinical Trials 28(3):316-23

[5] Eldridge S1, Kerry S, Torgerson DJ. Bias in identifying and recruiting participants in cluster randomised trials: what can be done? BMJ 2009;339:b4006

[6] Sarah J. Arnup, Andrew B. Forbes,1 Brennan C. Kahan,2 Katy E. Morgan,3 and Joanne E. McKenzie. The quality of reporting in cluster randomised crossover trials: proposal for reporting items and an assessment of reporting quality. Trials. 2016 Dec 6;17(1):575

[7] Kathleen Kiernan. Insights into Using the GLIMMIX Procedure to Model Categorical Outcomes with Random Effects. SAS Institute Inc. Paper SAS2179-2018

[8] Hertz-Picciotto I1, Rockhill B. Validity and efficiency of approximation methods for tied survival times in Cox regression. Biometrics. 1997 Sep;53(3):1151-6.

[9] Sveriges Farmaceuter. Lönestatistik 2018. Stockholm, Sweden: : Sveriges Farmaceuter 2019.

[10] Efron B, Tibshirani R. An Introduction to the Bootstrap. New York, NY: Chapman & Hall; 1993.

[11] Schafer JL. Multiple imputation: a primer. Statistical methods in medical research. 1999;8:3-15.
