# Supplementary material for: Effects of Hospital-Based Comprehensive Medication Reviews Including Postdischarge Follow-up on Older Patients’ Use of Health Care: A Cluster Randomized Clinical Trial
Source: JAMA Netw Open. 2021 Apr 30;4(4):e216303. doi: 10.1001/jamanetworkopen.2021.6303 (PMC8087955; doi:10.1001/jamanetworkopen.2021.6303)
Supplement: Supplement 3. — eMethods 1. Information From Trial Protocol and Statistical Analysis Plan eMethods 2. Sensitivity Analysis eTable 1. Primary Outcome Analysis for the Intention-to-Treat Population With Missing Data Imputed Under the Missing at Random Assumption eTable 2. Primary Outcome Analysis for the Intention-to-Treat Population With Missing Data Imputed Under the Missing Not at Random Assumption eTable 3. Secondary Outcomes Within 30 Days and 3, 6, and 12 Months eTable 4. Costs of Hospital-Based Care Within 6 and 12 Months eTable 5. Tests for Interaction Between Treatment Groups and Predefined Subgroups eFigure 1. Forest Plots for Age Subgroup Comparisons eFigure 2. Forest Plots for Number of Unplanned Hospital Visits Within 12 Months Before Inclusion Subgroups Comparisons eFigure 3. Forest Plot for Number of Prescribed Medications Upon Admission Subgroup Comparisons eFigure 4. Forest Plot for Use of an Automated Drug-Dispensing System in the Home Care Situation Subgroup Comparisons eFigure 5. Forest Plot for Previously Diagnosed Congestive Heart Failure Subgroup Comparisons eFigure 6. Forest Plot for Previously Diagnosed Chronic Obstructive Pulmonary Disease Subgroup Comparisons eFigure 7. Forest Plot for Previously Diagnosed Diabetes Mellitus Subgroup Comparisons eReferences. [file jamanetwopen-e216303-s003.pdf]

Supplementary Online Content

Kempen TGH, Bertilsson M, Hadziosmanovic N, et al. Effects of hospital-based comprehensive medication reviews including postdischarge follow-up on older patients' use of health care: a cluster randomized clinical trial. *JAMA Netw Open*. 2021;4(4):e216303. doi:10.1001/jamanetworkopen.2021.6303

eMethods 1. Information From Trial Protocol and Statistical Analysis Plan

eMethods 2. Sensitivity Analysis

eTable 1. Primary Outcome Analysis for the Intention-to-Treat Population With Missing Data Imputed Under the Missing at Random Assumption

eTable 2. Primary Outcome Analysis for the Intention-to-Treat Population With Missing Data Imputed Under the Missing Not at Random Assumption

eTable 3. Secondary Outcomes Within 30 Days and 3, 6, and 12 Months

eTable 4. Costs of Hospital-Based Care Within 6 and 12 Months

eTable 5. Tests for Interaction Between Treatment Groups and Predefined Subgroups

eFigure 1. Forest Plots for Age Subgroup Comparisons

eFigure 2. Forest Plots for Number of Unplanned Hospital Visits Within 12 Months Before Inclusion Subgroups Comparisons

eFigure 3. Forest Plot for Number of Prescribed Medications Upon Admission Subgroup Comparisons

eFigure 4. Forest Plot for Use of an Automated Drug-Dispensing System in the Home Care Situation Subgroup Comparisons

eFigure 5. Forest Plot for Previously Diagnosed Congestive Heart Failure Subgroup Comparisons

eFigure 6. Forest Plot for Previously Diagnosed Chronic Obstructive Pulmonary Disease Subgroup Comparisons

eFigure 7. Forest Plot for Previously Diagnosed Diabetes Mellitus Subgroup Comparisons

eReferences.

This supplementary material has been provided by the authors to give readers additional information about their work.

© 2021 Kempen TGH et al. *JAMA Network Open*.

eMethods 1. Information From Trial Protocol and Statistical Analysis Plan

The supplementary information given in this section is based on the Trial Protocol and Statistical Analysis Plan (Supplement 1)

Randomization

Crossover and randomization took place at cluster (ward) level within each hospital. Each ward participated in the trial for six consecutive eight-week study periods, which were divided into two separate blocks of three study periods each. During each period, one of three treatments (intervention 1, 2, or control) was provided at the ward, with permuted block randomization ensuring that each treatment was performed within each block. The randomized sequence was generated at Uppsala Clinical Research Center using SAS software (SAS Institute Inc). The computer-generated codes were held by the statistician to assure allocation concealment until the moment of randomization. Changes over time, such as seasonal differences, and any significant changes to the cluster setting during the study's inclusion period, such as an outbreak of multi-resistant bacteria at one of the study wards, can also influence the study outcomes. To account for any of these temporal effects, this process was replicated to create another three-period sequence per ward. As a result, each treatment was performed for two eight-week periods per ward. Study breaks, i.e., one or more consecutive weeks during which no patients were recruited, were planned for holiday periods, based on the expected lower availability of clinical pharmacist resources.

Primary and Secondary Outcome Measures

The primary outcome measure was the incidence of all-cause unplanned hospital visits (admissions plus visits to the emergency department (ED) within 12 months after index admission. An unplanned visit was defined as a visit which had not been part of the patient's treatment plan but resulted from an acute health problem. Secondary outcome measures were: the separate incidence rates of all-cause unplanned hospital admissions, ED visits, unplanned medication-related admissions, and primary care clinician (PCC) visits within 30 days, 3, 6, and 12 months; the incidence rates of unplanned hospital visits within 30 days and 3 and 6 months; the time from hospital discharge to first unplanned hospital visit within 12 months; all-cause mortality rates after 30 days, 3, 6, and 12 months; and costs of hospital-based care (total costs of all-cause unplanned hospital visits plus costs of interventions) within 6 and 12 months.

All primary and secondary outcomes are measurements of patients' healthcare utilization. Unplanned hospital visits is an objective and clinically important outcome measure reflecting the patients' morbidity. The interventions were expected to only influence medication-related hospital visits instead of all-cause hospital visits. However, due to the subjective nature of this outcome measure, we have chosen this as a secondary outcome measure. Because of a lack of available diagnostic information, it was deemed impossible to assess to what extent emergency department visits were medication related. Unplanned medication-related hospital admissions was therefore chosen as an outcome measure. Next to that, PCC visits was added as a relevant outcome measure for primary care practices. As this endpoint is not of similar clinical and economical importance as hospital visits, we considered it unsuitable to combine both into one composite primary endpoint. We pre-specified *unplanned* PCC visits as a secondary outcome, but reliable differentiation between planned and unplanned PCC visits proved impossible. The outcome was therefore changed to all PCC visits, prior to database closure. With time to first unplanned hospital visit we would be able to study effectiveness without the risk of contamination in terms of interventions provided to individual participants; e.g. a patient included as a control group patient being readmitted to hospital might receive a comprehensive medication review (CMR). We included costs of hospital-based care as a cost-effectiveness component in order to be able to value the interventions economically. Finally, all-cause mortality was added as our clinically most relevant endpoint, considering that our study might not be sufficiently powered to show a significant difference.

We have chosen the 30-day and 12-month follow-up periods in both the primary and secondary outcome measures to investigate the effectiveness on a short and mid-long term. These cut-off points

© 2021 Kempen TGH et al. *JAMA Network Open*.

are frequently used in clinical trials, for example in our previous randomized controlled trial (RCT), which allows for between study comparisons.[1,2] Next to that, 30-day hospital readmissions is an important measure for the quality of healthcare in many countries, e.g. Sweden and the USA.[2,3] In addition, we consider three and six months as logical follow-up periods in order to be able to show trends from 30 days to 12 months follow-up.

Pre-specified Subgroups

The primary outcome was also applied to pre-defined subgroups based on characteristics at index admission (baseline): age (65–74 years vs. ≥ 75 years); number of unplanned hospital visits within 12 months prior to inclusion (0–1 vs. > 1 visits); number of prescribed medications upon admission (< 5 vs. 5–9 vs. ≥ 10 medications); use of an automated drug dispensing system in the home care situation (yes vs. no); previously diagnosed congestive heart failure (HF, yes vs. no), chronic obstructive pulmonary disease (COPD, yes vs. no), and diabetes mellitus (DM, yes vs. no).

We defined these subgroups in order to be able to investigate which patients benefit the most from the interventions. According to Swedish legislation, all patients aged 75 years or older with five or more prescribed medications in use should receive a medication reconciliation with every hospital visit and, if deemed necessary, a CMR.[4] We therefore wanted to investigate whether there is a difference between patients aged 65-74 years and 75 or older in terms of effectiveness. The lower cut off point for the number of prescribed medications (<5 vs. 5-9) is also based on this legislation, whereas the other cut off point (≥10) is arbitrarily chosen based on our pilot study data: approximately 50% of the included patients used ten or more medications upon admission. Next to that, the number of previous unplanned hospital visits was considered an important predictor of our primary outcome (unplanned hospital visits). Based on our previous RCT and pilot study, the median number of previous unplanned hospital visits was expected to be one, motivating our cut off point (>1 visits). Lastly, HF, COPD and DM are seen as indications for which medication treatment plays such an essential role that any hospital admission related to these diseases are classified as preventable medication related hospital admissions in Sweden.[5] As the interventions in this study aimed to optimize medication treatment, we expected to see different effectiveness in participants who had previously been diagnosed with specifically these diseases.

Data Collection

Baseline data and data on admissions, visits, and mortality were extracted from each patient's electronic health record and the counties' healthcare registries, respectively. We pre-specified unplanned PCC visits as a secondary outcome, but reliable differentiation between planned and unplanned PCC visits proved impossible. The outcome was therefore changed to all PCC visits, prior to database closure. Costs of hospital-based care were retrieved from the counties' costs per patient system.[6] The average cost of each intervention was based on time measurements of pharmacists' work during the different study periods and an average clinical pharmacist hourly rate derived from the salary statistics of the Swedish Pharmacists Association for 2018.27 Unplanned hospital admissions were assessed by two final-year undergraduate pharmacy students with a validated method to identify unlikely or possibly medication-related admissions (AT-HARM10).[7] In case of doubt, an experienced clinical pharmacist was available to have the deciding vote. All possibly medication-related admissions were used as secondary outcome. All primary and secondary outcome data collection and assessments were blinded to treatment allocation.

Sample Size and Power Calculation

The proposed cluster randomized crossover design would result in an approximately 1:1:1 ratio of study participants in I1, I2 and in the control group. In our previous study in which we compared a comprehensive medication review with usual care at two hospital wards, the reduction in hospital visits was 16%.[8] Due to the multicenter nature of the current study, as well as an expected 20% of the patients revisiting study wards and receiving one or more additional study interventions (possibly diluting the estimated difference between groups), the expected reduction in this study was approximately 10%. Based on our previous RCT and data from our pilot study, we expected an

© 2021 Kempen TGH et al. *JAMA Network Open*.

incidence of two hospital visits (per patient year) in the control group. This meant that a 10% reduction would result in Number Needed to Treat of five to prevent one hospital visit during the 12 months follow-up, which we considered highly clinically relevant.

Power simulations were performed using the R package clusterPower version 0.5.[9] The power simulations were based on a fixed effects Poisson regression with a between cluster variance of 0.5. Analyses at the cluster level was assumed, even though the subsequent analyses would be performed at the individual level. We used anticipated cluster sizes from the pilot study (i.e. varying cluster sizes), with eight clusters and six periods per cluster in total. The expected mean number of at-risk days per patient was 290 and we assumed seven hospital visits per 1000 patient-days in the control group. With these assumptions, 2310 study participants in total would be needed to show a 10% reduction of hospital visits between I2 and the control group with a power of approximately 83% (α=5%). The corresponding power for an expected 3% difference between I1 and I2 would be approximately 48%. No compensation for withdrawals was accounted for in the sample size estimation, since the primary analysis would be based on the modified intention-to-treat principle.

Statistical Analysis

The differences in incidence rates of visits and admissions between treatment groups were compared using log-linear models with Poisson variance function in the framework of generalized linear mixed models, with adjustments made for cluster and period effects. The number of unplanned hospital visits within 12 months before index admission was used as a patient-level covariate, and the number of out-of-hospital-days was used as offset. Pairwise comparisons of all three treatment groups were of interest, as they could provide additional understanding of the effectiveness of CMR plus follow-up in relation to CMR only. We therefore deviated from the original trial protocol, where we proposed a hierarchical testing procedure, prior to database closure. Tukey's adjusted P values and 95% CIs were calculated to prevent multiple testing problems. The SAS procedure GLIMMIX (SAS Institute Inc) was used.[10,11] To account for overdispersion, a multiplicative overdispersion parameter was added to the variance function in all GLIMMIX models. Prespecified subgroups were analyzed with the same method as in the primary analysis, and a test for interaction with multivariable models was used to evaluate for statistically significant subgroup differences. Time to first unplanned hospital visit and mortality were analyzed with nested frailty models including gamma-distributed random effect. Study period was included as a fixed effect, cluster as a random effect, and the number of unplanned hospital visits within 12 months before index admission as a patient-level covariate. The non-parametric bootstrap method was used to compare costs of hospital-based care and to estimate 95% CIs. Pearson's chi-squared test was performed for differences in the percentage of dropouts between treatment groups, to assess potential recruitment bias.

A significance level of 0.05 (two-tailed) was used for all comparisons. All analyses were performed using SAS software (SAS Institute Inc). Statisticians were blinded to treatment allocation until database closure.

© 2021 Kempen TGH et al. *JAMA Network Open*.

eMethods 2. Sensitivity Analysis

The supplementary information given in this section is based on the Statistical Analysis Plan (Supplement 1)

Methods

The planned sensitivity analysis (per-protocol) was not performed, because of a perceived high risk of bias in the as-treated analysis, i.e., multimorbid patients were more likely to have received the intervention. The intention-to-treat (ITT) population (n = 2993) was therefore used for sensitivity analysis of the primary outcome measure, instead of the modified ITT population (n = 2637). The number of unplanned hospital visits was imputed for patients who did not provide informed consent (n = 349) and those who withdrew their consent before data collection (n = 7). We used a multiple imputation method based on study period, cluster, and planned treatment group. The SAS functions PROC MI and PROC MIANALYZE (SAS Institute Inc) were used to generate and combine ten imputed datasets. Two sensitivity analyses were performed: one in accordance with the missing at random (MAR) assumption, and one in accordance with the missing not at random (MNAR) assumption. With the MNAR assumption, imputed values for observations in the control group were adjusted using the shift parameter. In this way, the expected mean primary outcome of the control group was higher than that of observed mean value. We investigated which shift parameter reversed the statistical significance in the CMR plus follow-up (intervention 2) versus usual care (control) comparison. In the sensitivity analyses, no adjustments were made for the number of out-of-hospital days or the number of unplanned hospital visits in the 12 months before index admission, because these data were not available for these patients.

Results

With the MAR assumption, CMR plus follow-up increased the incidence of unplanned hospital visits within 12 months compared with usual care (rate ratio, 1.13; 95% CI, 1.04 to 1.22, eTable 1). With the MNAR assumption, a mean of 2.65 unplanned hospital visits per year (shift parameter 1.3) in the modified intention-to-treat population, instead of 1.59 visits per year, would reverse the difference between CMR plus follow-up and usual care from significant to non-significant (eTable 2).

© 2021 Kempen TGH et al. *JAMA Network Open*.

eTable 1. Primary Outcome Analysis for the Intention-to-Treat Population With Missing Data Imputed Under the Missing at Random Assumption

Comparison	Adjusted rate ratio[a] (95% CI)
CMR vs. CMR plus follow-up	0.94 (0.87 to 1.01)
CMR vs. usual care	1.06 (0.98 to 1.14)
CMR plus follow-up vs. usual care	1.13 (1.04 to 1.22)

Abbreviations: CMR = comprehensive medication review. [a]Estimates adjusted for study period, cluster and study intervention, but not for number of out-of-hospital days or number of unplanned hospital visits in the 12 months before index admission.

eTable 2. Primary Outcome Analysis for the Intention-to-Treat Population With Missing Data Imputed Under the Missing Not at Random Assumption

Shift parameter	Treatment group, mean imputed number of unplanned hospital visits[a]			Adjusted rate ratio[b] (95% CI)	
	CMR (n = 95)	CMR plus follow-up (n = 119)	Usual care (n = 142)	CMR vs. usual care	CMR plus follow-up vs. usual care
0	1.65	1.80	1.67	1.07 (0.99 to 1.16)	1.15 (1.07 to 1.24)
0.1	1.65	1.80	1.74	1.06 (0.98 to 1.15)	1.15 (1.07 to 1.24)
0.2	1.65	1.80	1.81	1.06 (0.98 to 1.15)	1.14 (1.06 to 1.23)
0.3	1.65	1.80	1.87	1.06 (0.97 to 1.14)	1.14 (1.06 to 1.23)
0.4	1.65	1.80	1.94	1.05 (0.97 to 1.14)	1.13 (1.05 to 1.22)
0.5	1.65	1.80	2.02	1.04 (0.96 to 1.13)	1.12 (1.04 to 1.21)
0.6	1.65	1.80	2.11	1.04 (0.96 to 1.13)	1.12 (1.04 to 1.21)
0.7	1.65	1.80	2.19	1.03 (0.95 to 1.12)	1.11 (1.03 to 1.20)
0.8	1.65	1.80	2.26	1.03 (0.95 to 1.12)	1.11 (1.03 to 1.19)
0.9	1.65	1.80	2.34	1.02 (0.94 to 1.11)	1.10 (1.02 to 1.19)
1.0	1.65	1.80	2.42	1.02 (0.94 to 1.10)	1.10 (1.02 to 1.18)
1.1	1.65	1.80	2.50	1.01 (0.93 to 1.10)	1.09 (1.01 to 1.17)
1.2	1.65	1.80	2.58	1.01 (0.93 to 1.09)	1.08 (1.01 to 1.17)
1.3	1.65	1.80	2.65	1.00 (0.92 to 1.09)	1.08 (1.00 to 1.16)[c]
1.4	1.65	1.80	2.73	1.00 (0.92 to 1.08)	1.07 (1.00 to 1.16)
1.5	1.65	1.80	2.83	0.99 (0.91 to 1.08)	1.07 (0.99 to 1.15)

Abbreviations: CMR = comprehensive medication review. [a]The corresponding mean numbers of unplanned hospital visits for the modified intention-to-treat population per treatment group: CMR (n = 922): 1.70, CMR plus follow-up (n = 823): 1.91, usual care (n = 892): 1.59. [b]Estimates adjusted for study period, cluster, and study intervention, but not for number of out-of-hospital days or number of unplanned hospital visits in the 12 months before index admission. [c]p = 0.048, the mean value of imputed unplanned hospital visits is adjusted using a shift parameter from 0.0 to 1.5; the shift value that reverses the statistical significance of the comprehensive medication review plus follow-up group versus usual care comparison is 1.3.

© 2021 Kempen TGH et al. *JAMA Network Open*.

eTable 3. Secondary Outcomes Within 30 Days and 3, 6, and 12 Months

Outcome	Treatment group, crude rate			Crude rate ratio (95% CI)		Adjusted ratio[a] (95% CI[b])	
	CMR (n=922)	CMR plus follow-up (n=823)	Usual care (n=892)	CMR vs usual care	CMR plus follow-up vs usual care	CMR vs usual care	CMR plus follow-up vs usual care
Unplanned hospital visits within 30 days	0.30	0.29	0.29	1.05 (0.88 to 1.25)	1.01 (0.84 to 1.21)	1.02 (0.71 to 1.45)	0.96 (0.66 to 1.39)
Unplanned hospital visits within 3 months	0.69	0.77	0.62	1.11 (0.99 to 1.25)	1.25 (1.11 to 1.40)	1.09 (0.87 to 1.36)	1.20 (0.96 to 1.49)
Unplanned hospital visits within 6 months	1.08	1.18	0.99	1.09 (1.00 to 1.20)	1.20 (1.09 to 1.32)	1.06 (0.89 to 1.27)	1.15 (0.96 to 1.37)
ED visits within 30 days	0.13	0.13	0.11	1.20 (0.92 to 1.57)	1.20 (0.91 to 1.58)	1.17 (0.73 to 1.89)	1.13 (0.70 to 1.85)
ED visits within 3 months	0.30	0.38	0.26	1.13 (0.95 to 1.35)	1.46 (1.23 to 1.73)	1.11 (0.82 to 1.49)	1.39 (1.04 to 1.85)
ED visits within 6 months	0.48	0.58	0.43	1.13 (0.98 to 1.30)	1.35 (1.18 to 1.55)	1.10 (0.87 to 1.41)	1.27 (1.00 to 1.62)
ED visits within 12 months	0.84	0.97	0.71	1.18 (1.06 to 1.31)	1.36 (1.22 to 1.51)	1.16 (0.94 to 1.44)	1.29 (1.05 to 1.59)
Unplanned hospital admissions within 30 days	0.17	0.16	0.18	0.95 (0.76 to 1.20)	0.89 (0.70 to 1.13)	0.92 (0.61 to 1.39)	0.85 (0.55 to 1.32)
Unplanned hospital admissions within 3 months	0.39	0.39	0.35	1.10 (0.94 to 1.28)	1.09 (0.93 to 1.28)	1.08 (0.84 to 1.38)	1.06 (0.82 to 1.37)
Unplanned hospital admissions within 6 months	0.59	0.60	0.56	1.06 (0.94 to 1.20)	1.08 (0.95 to 1.23)	1.03 (0.84 to 1.26)	1.05 (0.86 to 1.29)
Unplanned hospital admissions within 12 months	0.89	0.98	0.91	0.98 (0.89 to 1.08)	1.08 (0.98 to 1.19)	0.95 (0.80 to 1.12)	1.04 (0.88 to 1.24)
Unplanned medication-related admissions within 30 days	0.06	0.07	0.07	0.84 (0.58 to 1.21)	1.03 (0.72 to 1.48)	0.85 (0.46 to 1.54)	1.02 (0.57 to 1.84)
Unplanned medication-related admissions within 3 months	0.14	0.15	0.14	0.95 (0.74 to 1.22)	1.03 (0.80 to 1.33)	0.94 (0.64 to 1.37)	1.04 (0.71 to 1.53)
Unplanned medication-related admissions within 6 months	0.21	0.21	0.22	0.96 (0.78 to 1.17)	0.98 (0.80 to 1.21)	0.93 (0.69 to 1.26)	0.98 (0.72 to 1.34)
Unplanned medication-related admissions within 12 months	0.29	0.36	0.32	0.92 (0.78 to 1.09)	1.13 (0.96 to 1.33)	0.89 (0.69 to 1.16)	1.12 (0.87 to 1.45)
PCC visits within 30 days	0.60	0.51	0.57	1.05 (0.93 to 1.19)	0.91 (0.80 to 1.04)	1.03 (0.85 to 1.26)	0.94 (0.75 to 1.17)

© 2021 Kempen TGH et al. *JAMA Network Open*.

Outcome	Treatment group, crude rate			Crude rate ratio (95% CI)		Adjusted ratio[a] (95% CI[b])	
	CMR (n=922)	CMR plus follow-up (n=823)	Usual care (n=892)	CMR vs usual care	CMR plus follow-up vs usual care	CMR vs usual care	CMR plus follow-up vs usual care
PCC visits within 3 months	1.55	1.36	1.39	1.11 (1.03 to 1.20)	0.98 (0.90 to 1.07)	1.11 (0.95 to 1.30)	1.03 (0.87 to 1.22)
PCC visits within 6 months	2.65	2.34	2.44	1.09 (1.02 to 1.15)	0.96 (0.90 to 1.02)	1.08 (0.93 to 1.25)	1.01 (0.86 to 1.18)
PCC visits within 12 months	4.43	4.02	4.25	1.04 (1.00 to 1.09)	0.95 (0.90 to 0.99)	1.04 (0.91 to 1.19)	0.99 (0.86 to 1.15)
All-cause mortality after 30 days, No. (%)	76 (8.2)	61 (7.4)	77 (8.6)	0.96[c] (0.70 to 1.31)	0.85[c] (0.61 to 1.19)	0.94[c] (0.68 to 1.29)	0.79[c] (0.57 to 1.11)
All-cause mortality after 3 months, No. (%)	127 (13.8)	108 (13.1)	118 (13.2)	1.04[c] (0.81 to 1.34)	0.98[c] (0.76 to 1.28)	1.02[c] (0.79 to 1.32)	0.95[c] (0.73 to 1.23
All-cause mortality after 6 months, No. (%)	171 (18.5)	151 (18.3)	153 (17.2)	1.08[c] (0.87 to 1.35)	1.06[c] (0.85 to 1.33)	1.07[c] (0.86 to 1.33)	1.02[c] (0.81 to 1.29)
All-cause mortality after 12 months, No. (%)	234 (25.4)	209 (25.4)	227 (25.4)	1.00[c] (0.83 to 1.20)	0.99[c] (0.82 to 1.20)	0.98[c] (0.81 to 1.18)	0.95[c] (0.79 to 1.15)

Abbreviations: CMR = comprehensive medication review; ED = emergency department; PCC = primary care clinician. [a]Estimates adjusted for cluster (ward) as random effect, study period as fixed effect, and unplanned hospital visits in 12 months prior to inclusion as patient-level covariate. [b]Tukey´s adjusted 95% confidence intervals for multiple comparisons. [c]Hazard ratio.

eTable 4. Costs of Hospital-Based Care Within 6 and 12 Months

				Difference of mean (95% CI[a])	
	CMR (n=922)	CMR plus follow-up (n=823)	Usual care (n=892)	CMR vs usual care	CMR plus follow-up vs usual care
Costs of hospital-based care within 6 months[b], mean (SD), $[c]	6023 (12 579)	6272 (14 601)	6046 (13 802)	-21 (-1246 to 1182)	226 (-1118 to 1574)
Costs of hospital-based care within 12 months[b], mean (SD), $[c]	8987 (17 121)	9981 (18 963)	9901 (18 464)	-914 (-2564 to 719)	55 (-1721 to 1823)

Abbreviation: CMR = comprehensive medication review. [a]Based on 100 000 bootstrap estimates. [b]Including intervention costs: $58 for CMR and $94 for CMR plus follow-up. [c]Based on a Swedish krona (SEK) to USD ($) conversion rate of 0.11246 (as per 1 January 2019).

eTable 5. Tests for Interaction Between Treatment Groups and Predefined Subgroups

Subgroup	P value
Age	0.11
Number of unplanned hospital visits within 12 months prior to inclusion	< 0.01
Number of prescribed medications upon admission	0.76
Use of an automated drug dispensing system in the home care situation	0.38
Previously diagnosed congestive heart failure	0.36
Previously diagnosed chronic obstructive pulmonary disease	0.14
Previously diagnosed diabetes mellitus	0.22

© 2021 Kempen TGH et al. *JAMA Network Open*.

eFigure 1. Forest Plots for Age Subgroup Comparisons

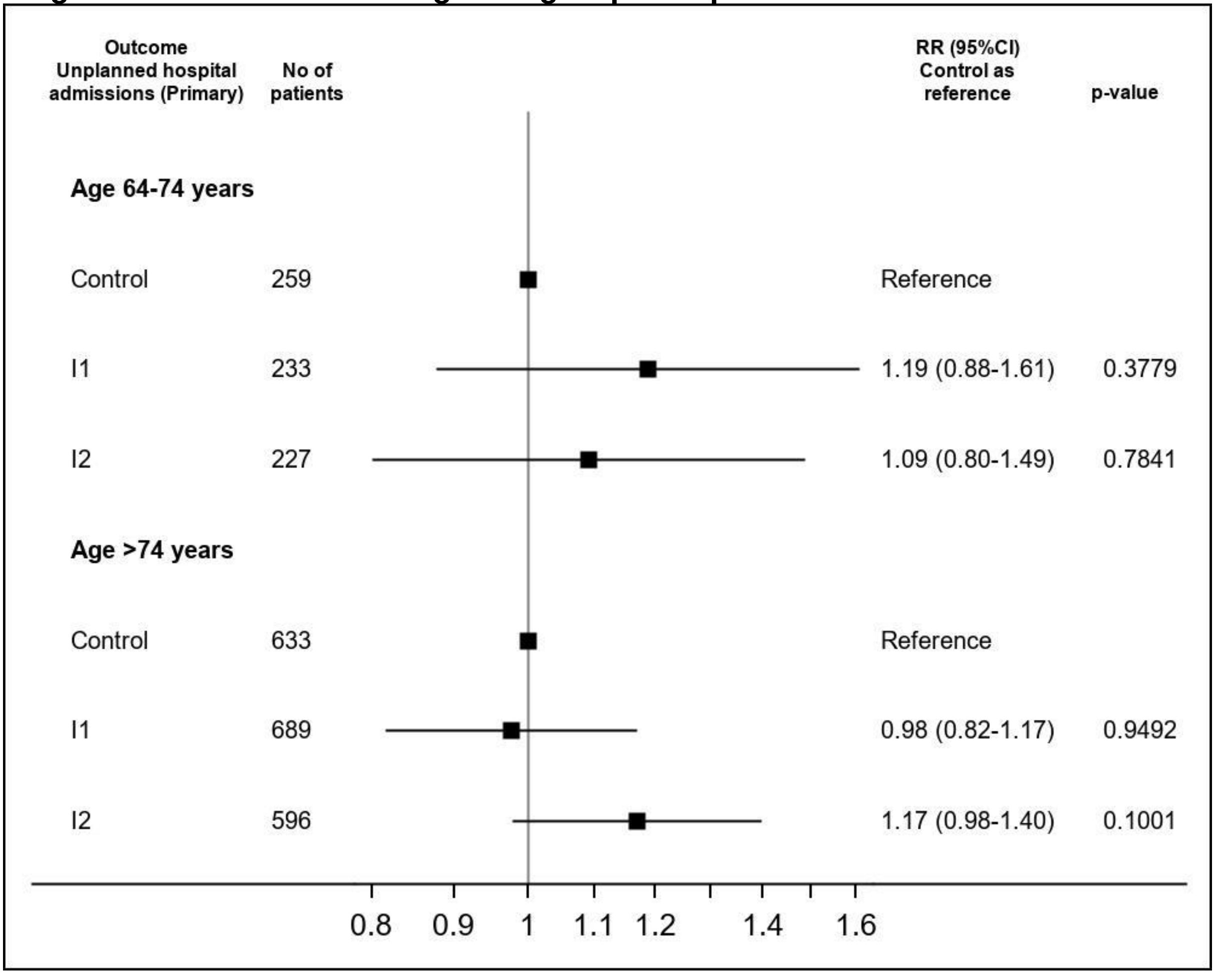

Estimates adjusted for cluster (ward) effect and study period effect, unplanned hospital visits 12 months prior to inclusion as patient level covariate, and number of out-of-hospital-days as offset. Tukey´s adjusted p values and 95% confidence intervals for multiple comparisons.
Abbreviations: I1 = intervention 1 (comprehensive medication review); I2 = intervention 2 (comprehensive medication review plus follow-up); RR = rate ratio

© 2021 Kempen TGH et al. *JAMA Network Open*.

eFigure 2. Forest Plots for Number of Unplanned Hospital Visits within 12 Months Before Inclusion Subgroups Comparisons

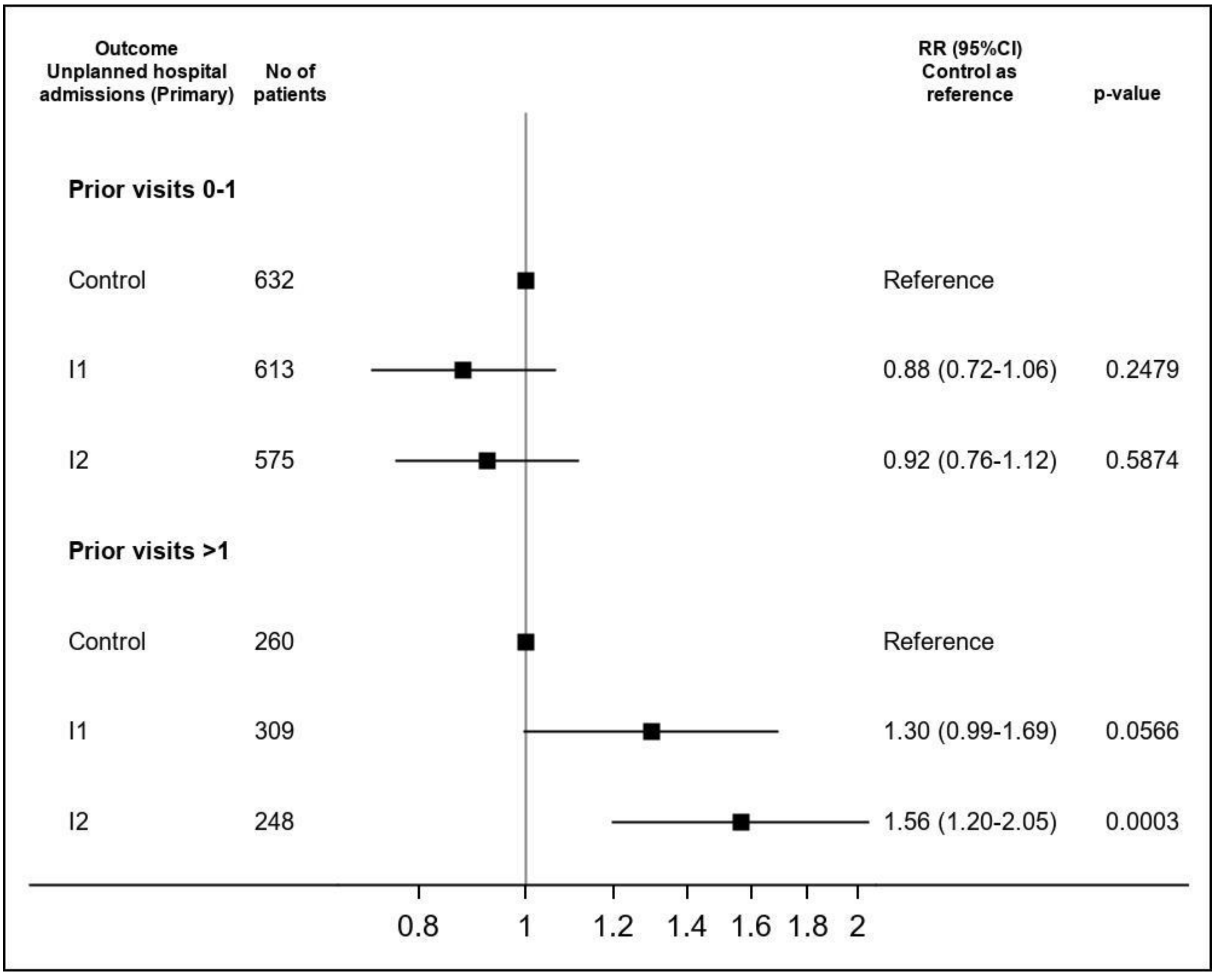

Estimates adjusted for cluster (ward) effect and study period effect, and number of out-of-hospital-days as offset. Tukey´s adjusted P values and 95% CIs for multiple comparisons. Abbreviations: I1 = intervention 1 (comprehensive medication review); I2 = intervention 2 (comprehensive medication review plus follow-up); RR = rate ratio

© 2021 Kempen TGH et al. *JAMA Network Open*.

eFigure 3. Forest Plot for Number of Prescribed Medications Upon Admission Subgroup Comparisons

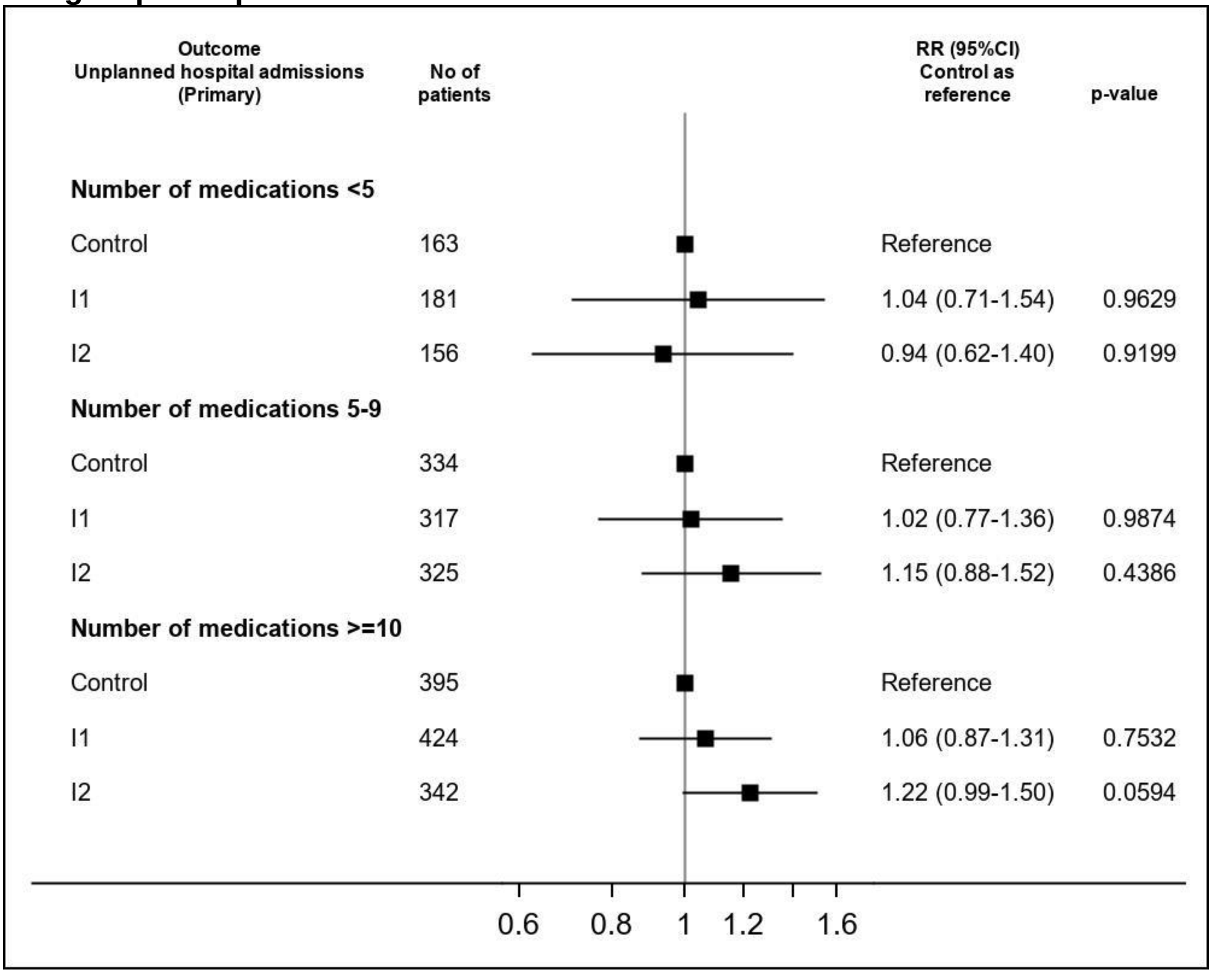

Estimates adjusted for cluster (ward) effect and study period effect, unplanned hospital visits 12 months prior to inclusion as patient level covariate, and number of out-of-hospital-days as offset. Tukey´s adjusted P values and 95% CIs for multiple comparisons. Abbreviations: I1 = intervention 1 (comprehensive medication review); I2 = intervention 2 (comprehensive medication review plus follow-up); RR = rate ratio

© 2021 Kempen TGH et al. *JAMA Network Open*.

eFigure 4. Forest Plot for Use of an Automated Drug-Dispensing System in the Home Care Situation Subgroup Comparisons

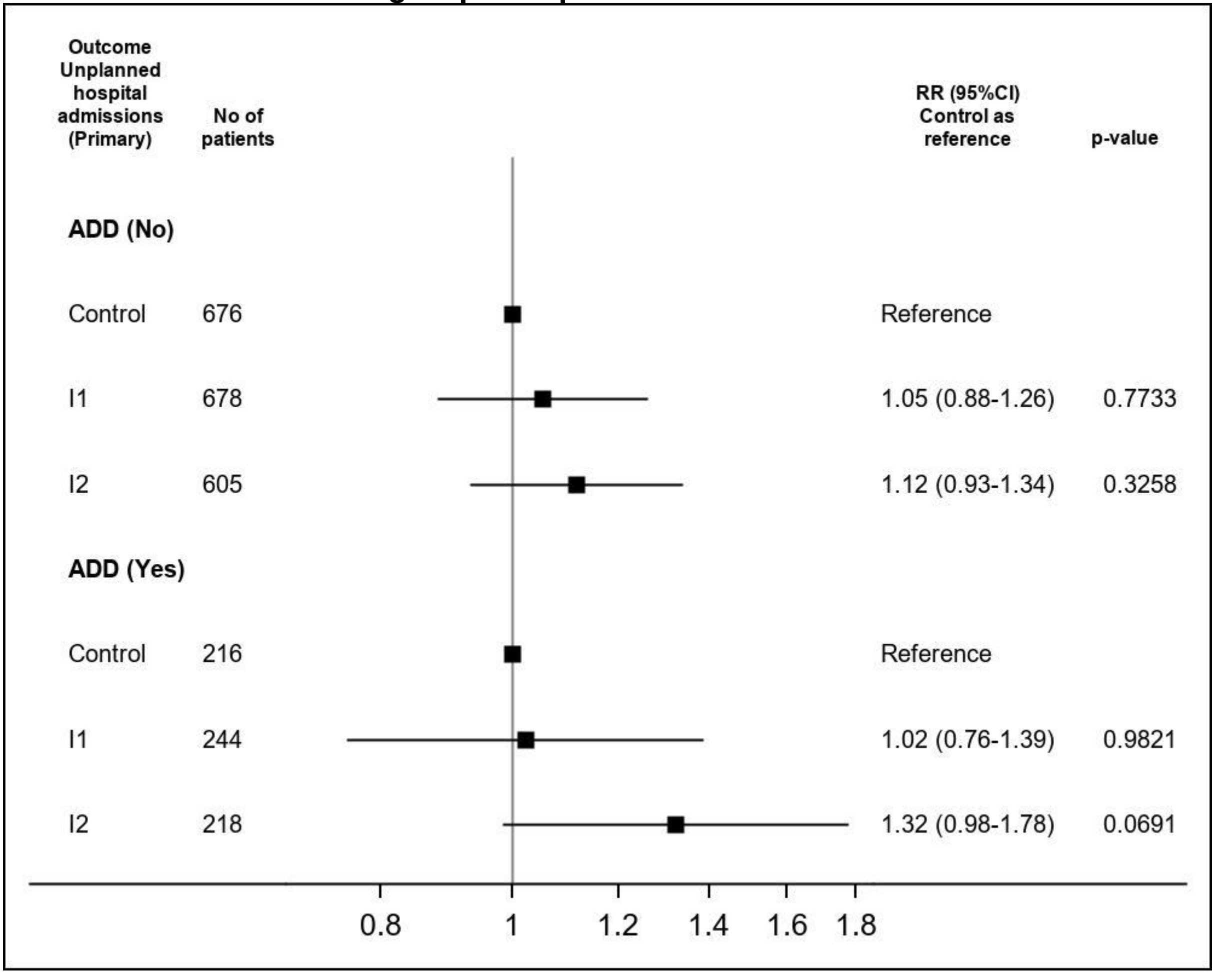

Estimates adjusted for cluster (ward) effect and study period effect, unplanned hospital visits 12 months prior to inclusion as patient level covariate, and number of out-of-hospital-days as offset. Tukey´s adjusted P values and 95% CIs for multiple comparisons. Abbreviations: ADD = automated drug dispensing; I1 = intervention 1 (comprehensive medication review); I2 = intervention 2 (comprehensive medication review plus follow-up); RR = rate ratio

© 2021 Kempen TGH et al. *JAMA Network Open*.

eFigure 5. Forest Plot for Previously Diagnosed Congestive Heart Failure Subgroup Comparisons

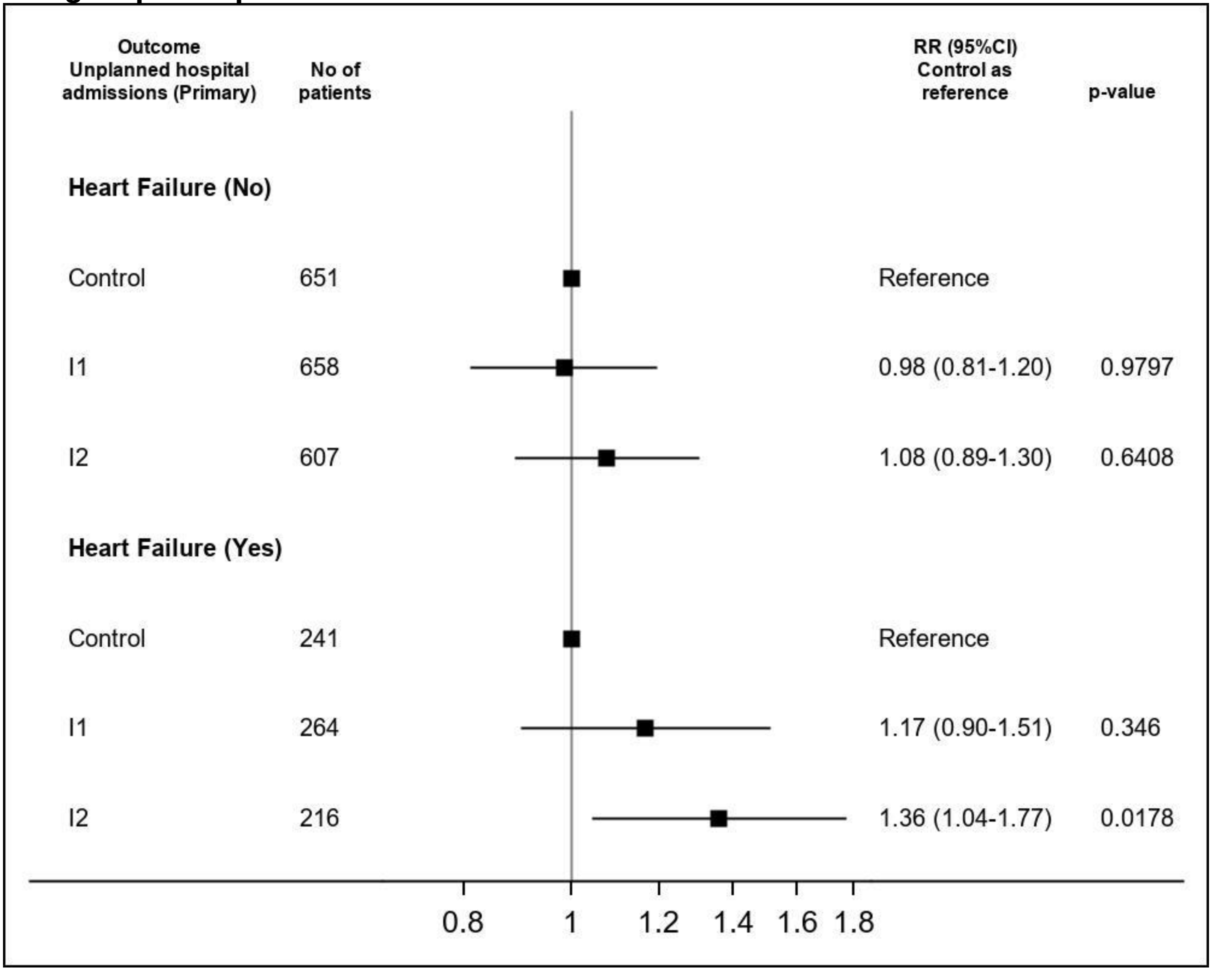

Estimates adjusted for cluster (ward) effect and study period effect, unplanned hospital visits 12 months prior to inclusion as patient level covariate, and number of out-of-hospital-days as offset. Tukey´s adjusted P values and 95% CIs for multiple comparisons. Abbreviations: I1 = intervention 1 (comprehensive medication review); I2 = intervention 2 (comprehensive medication review plus follow-up); RR = rate ratio

© 2021 Kempen TGH et al. *JAMA Network Open*.

eFigure 6. Forest Plot for Previously Diagnosed Chronic Obstructive Pulmonary Disease Subgroup Comparisons

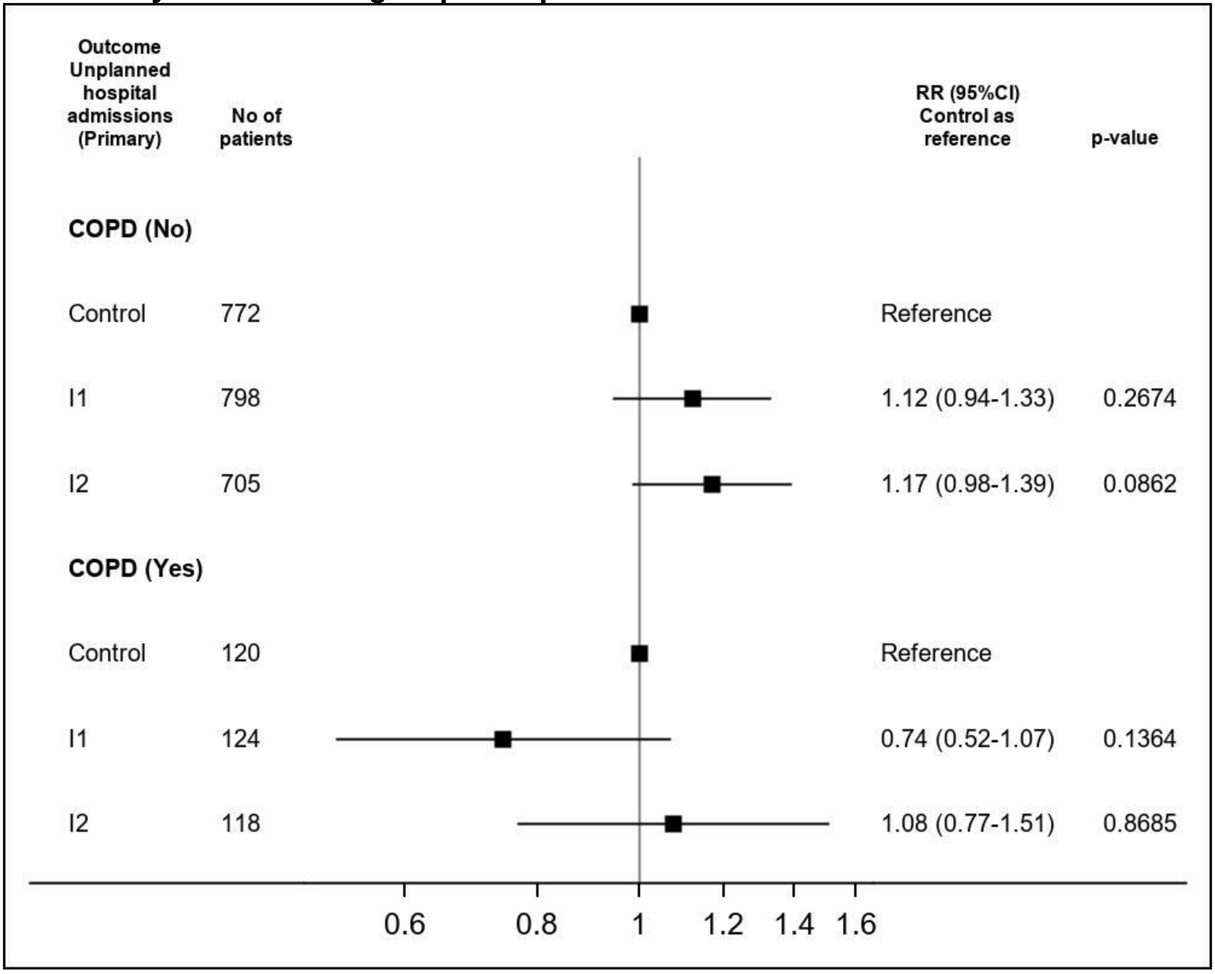

Estimates adjusted for cluster (ward) effect and study period effect, unplanned hospital visits 12 months prior to inclusion as patient level covariate, and number of out-of-hospital-days as offset. Tukey´s adjusted P values and 95% CIs for multiple comparisons. Abbreviations: I1 = intervention 1 (comprehensive medication review); I2 = intervention 2 (comprehensive medication review plus follow-up); RR = rate ratio

© 2021 Kempen TGH et al. *JAMA Network Open*.

eFigure 7. Forest Plot for Previously Diagnosed Diabetes Mellitus Subgroup Comparisons

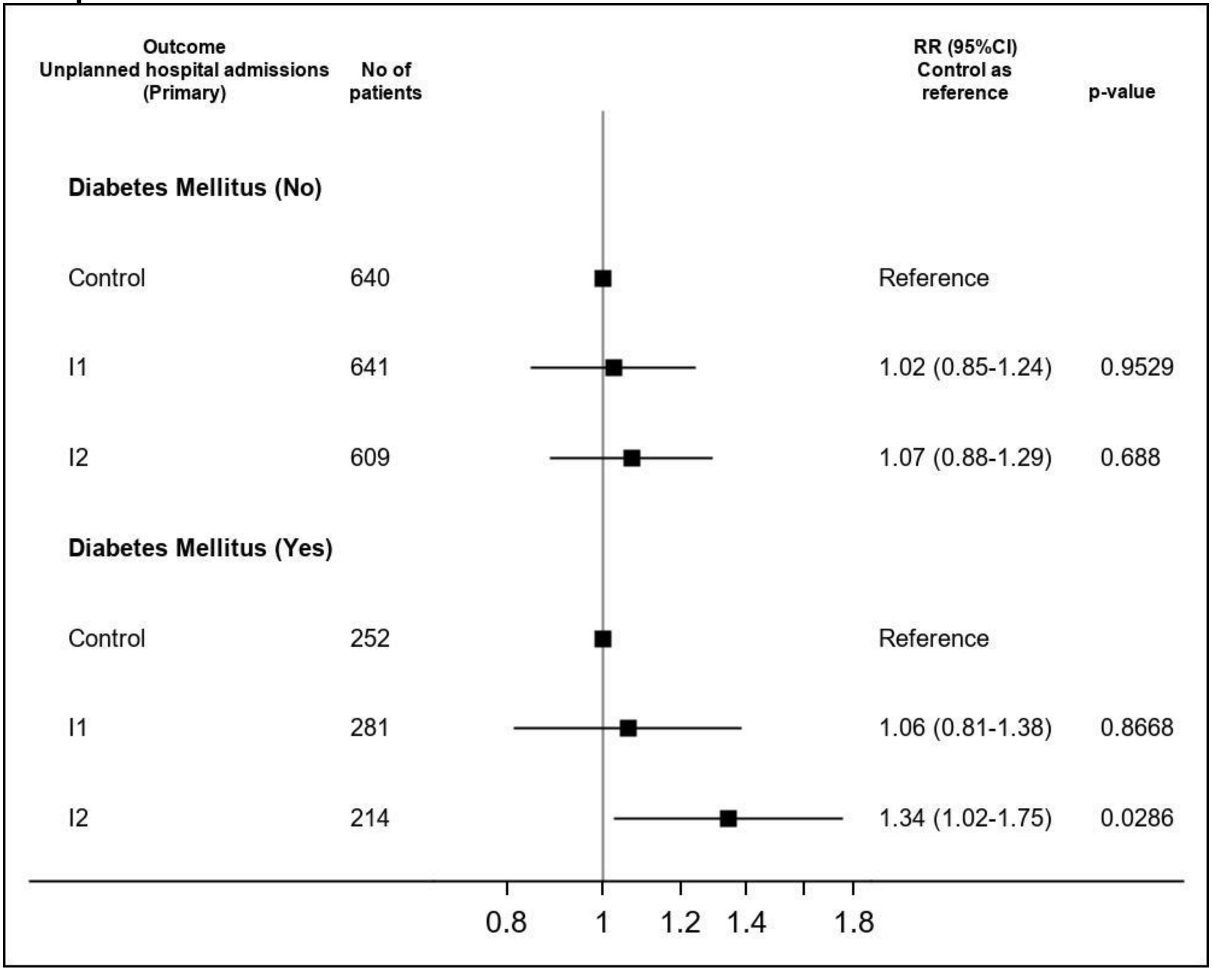

Estimates adjusted for cluster (ward) effect and study period effect, unplanned hospital visits 12 months prior to inclusion as patient level covariate, and number of out-of-hospital-days as offset. Tukey´s adjusted P values and 95% CIs for multiple comparisons. Abbreviations: I1 = intervention 1 (comprehensive medication review); I2 = intervention 2 (comprehensive medication review plus follow-up); RR = rate ratio

© 2021 Kempen TGH et al. *JAMA Network Open*.

eReferences

1. Christensen M, Lundh A. Medication review in hospitalised patients to reduce morbidity and mortality. *Cochrane Database Syst Rev*. 2016;CD008986. doi:10.1002/14651858.CD008986.pub3
2. Renaudin P, Boyer L, Esteve M-A, Bertault-Peres P, Auquier P, Honore S. Do pharmacist-led medication reviews in hospitals help reduce hospital readmissions? A systematic review and meta-analysis. *Br J Clin Pharmacol*. 2016;82(6):1660-1673. doi:10.1111/bcp.13085
3. Leppin AL, Gionfriddo MR, Kessler M, et al. Preventing 30-day hospital readmissions: a systematic review and meta-analysis of randomized trials. *JAMA Intern Med*. 2014;174(7):1095-1107. doi:10.1001/jamainternmed.2014.1608
4. Socialstyrelsen. *Ändring i Föreskrifterna Och Allmänna Råden (SOSFS 2000:1) Om Läkemedelshantering i Hälso- Och Sjukvården*. Stockholm, Sweden: National Board of Health and Welfare; 2012:1-11.
5. Socialstyrelsen. *Utveckling Av Indikatorerna Undvikbar Slutenvård Och Oplanerade Återinskrivningar*. Stockholm, Sweden: National Board of Health and Welfare; 2014.
6. Sveriges Farmaceuter. *Lönestatistik 2018*. Stockholm, Sweden: Sveriges Farmaceuter; 2019.
7. Kempen TGH, Hedström M, Olsson H, et al. Assessment tool for hospital admissions related to medications: development and validation in older patients. *Int J Clin Pharm*. 2019;41(1):198-206. doi:10.1007/s11096-018-0768-8
8. Gillespie U, Alassaad A, Henrohn D, et al. A Comprehensive Pharmacist Intervention to Reduce Morbidity in Patients 80 Years or Older: A Randomized Controlled Trial. *Arch Intern Med*. 2009;169:894-900. doi:10.1001/archinternmed.2009.71
9. Reich NG, Myers JA, Obeng D, Milstone AM, Perl TM. Empirical Power and Sample Size Calculations for Cluster-Randomized and Cluster-Randomized Crossover Studies. *PLoS One*. 2012;7(4):e35564. https://doi.org/10.1371/journal.pone.0035564.
10. Arnup SJ, Forbes AB, Kahan BC, Morgan KE, McKenzie JE. The quality of reporting in cluster randomised crossover trials: proposal for reporting items and an assessment of reporting quality. *Trials*. 2016;17(1):575. doi:10.1186/s13063-016-1685-6
11. Kiernan K, SAS Institute Inc. *Insights into Using the GLIMMIX Procedure to Model Categorical Outcomes with Random Effects*.; 2018. Paper SAS2179-2018.

© 2021 Kempen TGH et al. *JAMA Network Open*.
