# Supplementary material for: Effects of Hospital-Based Comprehensive Medication Reviews Including Postdischarge Follow-up on Older Patients’ Use of Health Care: A Cluster Randomized Clinical Trial
Source: JAMA Netw Open. 2021 Apr 30;4(4):e216303. doi: 10.1001/jamanetworkopen.2021.6303 (PMC8087955; doi:10.1001/jamanetworkopen.2021.6303)
Supplement: Supplement 4. — Data Sharing Statement [file jamanetwopen-e216303-s004.pdf]

Data Sharing Statement

Kempen. Effects of Hospital-Based Comprehensive Medication Reviews Including Postdischarge Follow-up on Older Patients' Use of Health Care. *JAMA Netw Open*. Published April 30, 2021. doi:10.1001/jamanetworkopen.2021.6303

Data
Data available: Yes
Data types: Deidentified participant data
How to access data: Requests to access deidentified individual participant data, informed consent forms, case report forms, standard operating procedures, or the data management plan should be addressed to the corresponding author at thomas.kempen@medsci.uu.se. All proposals requesting data access will need to specify an analysis plan and have approval from the MedBridge trial research group before data release.
When available: With publication

Supporting Documents
Document types: Other (please specify)
Additional Information: Deidentified individual participant data, informed consent forms, case report forms, standard operating procedures, and the data management plan
How to access documents: thomas.kempen@medsci.uu.se
When available: With publication

Additional Information
Who can access the data: Anyone requesting the data
Types of analyses: Any non-commercial research purposes
Mechanisms of data availability: All proposals requesting data access will need to specify an analysis plan and have approval from the MedBridge trial research group before data release.
